# Polar day syndrome: differences in growth, photosynthetic traits and sink-size patterns between northern and southern Finnish silver birch (*Betula pendula* Roth) provenances in native and non-native photoperiods

**DOI:** 10.1093/treephys/tpac104

**Published:** 2022-09-01

**Authors:** Antti Tenkanen, Markku Keinänen, Elina Oksanen, Sarita Keski-Saari, Sari Kontunen-Soppela

**Affiliations:** University of Eastern Finland, Department of Environmental and Biological Sciences, Yliopistokatu 7, P.O. Box 111, 80101 Joensuu, Finland; University of Eastern Finland, Department of Environmental and Biological Sciences, Yliopistokatu 7, P.O. Box 111, 80101 Joensuu, Finland; University of Eastern Finland, Institute of Photonics, Yliopistokatu 7, PO Box 111, 80101 Joensuu, Finland; University of Eastern Finland, Department of Environmental and Biological Sciences, Yliopistokatu 7, P.O. Box 111, 80101 Joensuu, Finland; University of Eastern Finland, Department of Environmental and Biological Sciences, Yliopistokatu 7, P.O. Box 111, 80101 Joensuu, Finland; University of Eastern Finland, Department of Environmental and Biological Sciences, Yliopistokatu 7, P.O. Box 111, 80101 Joensuu, Finland

**Keywords:** *Betula pendula* (silver birch), biomass allocation, continuous light, ecotype, gas exchange, growth rate, leaf longevity, photoperiod, photosynthesis, shoot:root ratio

## Abstract

Continuous light (CL) is available throughout the polar day for plants in the Arctic during the growing season, whereas provenances of the same species experience a very different environment with non-CL (NCL) just a few latitudes to the south. Both provenances need to acclimate to climate warming, yet we lack comprehensive understanding of how their growth, photosynthesis and leaf traits differ. Further, the provenances presumably have morphological and physiological adaptations to their native environments and therefore differ in response to photoperiod. We tested the height growth, leaf longevity, biomass accumulation, biomass allocation and rates of gas exchange of northern (67°N) and southern (61°N) Finnish silver birch (*Betula pendula* Roth) origins in CL- and NCL-treatments in a 4-month chamber experiment. Irrespective of photoperiod, 67°N had higher area-based photosynthetic rate (*A*_net_), stomatal conductance (*g*_s_) and relative height growth rate (RGR), but lower stomatal density and fewer branches and leaves than 61°N. Photoperiod affected height growth cessation, biomass and photosynthetic traits, whereas leaf longevity and many leaf functional traits remained unchanged. In CL, both provenances had lower *g*_s_, higher RGR, increased shoot:root ratio and increased sink sizes (more branching, more leaves, increased total plant dry weight) compared with NCL. In NCL, 67°N ceased height growth earlier than in CL, which altered biomass accumulation and distribution patterns. Northern conditions impose challenges for plant growth and physiology. Whether a provenance inhabits and is adapted to an area with or without CL can also affect its response to the changing climate. Northern birches may have adapted to CL and the short growing season with a ‘polar day syndrome’ of traits, including relatively high gas exchange rates with low leaf biomass and growth traits that are mainly limited by the environment and the earlier growth cessation (to avoid frost damage).

## Introduction

With the global climate facing drastic changes it is more important than ever to understand the differences in growth, photosynthesis and sink-size patterns of plant ecotypes exposed to different photoperiods at various latitudes. Plants from very high latitudes (above the Arctic circle, approximately 66°33′N), such as those from northernmost Finland, receive continuous light (CL) throughout the polar day. Continuous light supplies uninterrupted energy, but it also creates challenges for light-dependent signaling and can cause stress such as photosynthetic downregulation, senescence and photooxidative damage (Velez-Ramirez et al. 2011). However, all of these effects may depend on the received irradiance. Therefore, it is noteworthy that northern plants are also exposed to diurnal cycles of light and temperature, which may maintain their biological clocks (Velez-Ramirez et al. 2011, Fernández-Marín et al. 2018). Despite northern areas heating up faster than southern ones due to global warming (Jansen et al. 2020), exposing plants to various new combinations of higher temperatures and CL, there are still rather few studies on the effects of CL on northern tree species.

**Figure 1.**
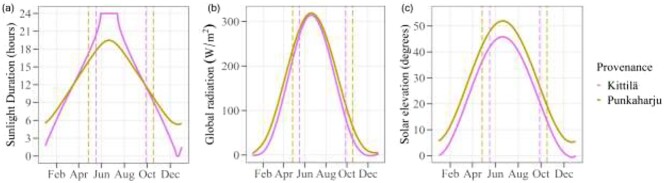
(a) Sunlight duration, (b) global radiation incident upon a horizontal surface and (c) solar elevation corrected for atmospheric refraction through 1 year in natural settings at Kittilä (67°44′N, 24°50′E) and Punkaharju (61°48′N, 29°19′E). Drawn from NOAA solar position and sunrise/sunset calculator data (NOAA 2022). Vertical dashed lines delimit the approximate maximum length of the average thermal growing season during 1991–2020 (with a threshold temperature of +5 °C) at Kittilä and Punkaharju according to FMI (Finnish Meteorological Institute 2022). For (a) and (c) data are daily values at noon. For (b) data are values at 15 min intervals. The relationship for summed daily irradiances is similar to that in (b), but the amount of incident radiation is larger.

Sun elevation angle affects the quality and quantity of radiation received at different latitudes (Figure 1c, Supplementary Methods S1 available as Supplementary data at *Tree Physiology* Online). In northern Finland, the daily averaged global radiation is always less than in the south, even during the annual period of CL (NOAA 2022). However, the difference in integrated irradiance between the north and the south is diminished during the growing season (Figure 1b, Supplementary Methods S1 available as Supplementary data at *Tree Physiology* Online, NOAA 2022). Considering the effects of light intensity is nevertheless relevant for experiments that utilize CL. Plants grown in high light (as in high total daily integrated irradiance, not just high peak intensity) often have leaves with higher leaf mass per area (LMA) (Poorter et al. 2009, Tullus et al. 2021), more nitrogen and higher photosynthetic rates per leaf area, but less chlorophyll on a mass basis (Lichtenthaler et al. 2007, Niinemets 2007) or on both a mass and area basis (Ellsworth and Reich 1992) than low-light leaves. In general, high-light phenotypes optimize photosynthetic capacity (and photoprotection), while low-light phenotypes optimize light capture (Valladares et al. 2012).

As leaves need to operate their main task of photosynthesis despite environmental conditions, structural and functional leaf traits can reveal differences in ecotype strategies. In the north, many leaf traits should be affected by adaptations to the cold. For example, thick and heavy leaves (high LMA) can be adaptive in the cold (Poorter et al. 2009). However, it is not entirely clear how much the light and temperature environments individually contribute to LMA, although light is often the predominant factor (Poorter et al. 2009, 2019). Nonetheless, an increase in LMA comes with an increase in building cost and can affect, e.g., leaf optics and gas conductance properties (Poorter et al. 2009, Tholen et al. 2012). Differences in LMA can therefore be related to the plant’s carbon balance and payback time for new leaves (Williams et al. 1989, Poorter et al. 2006). These effects can ultimately lead to adjustments in optimal leaf lifespan. Indeed, globally, leaf longevity correlates positively with mean annual temperature for deciduous species and negatively for evergreen species (Kikuzawa et al. 2013).

Leaf longevity also covaries with photosynthetic functional traits. Maximal carbon gain strategies predict that leaf lifespan should be intrinsically negatively connected to photosynthetic rate and positively to leaf construction cost (Reich et al. 1992, Kikuzawa 1995). Observed across species, maximum net photosynthetic rate (leaf mass-based *A*_mass_ and to a lesser extent area-based *A*_net_), area-based stomatal conductance (*g*_s_) and mass-based N decrease with increasing leaf longevity, while LMA increases with increasing leaf longevity (Reich et al. 1992). Therefore, environmental conditions favoring high LMA and leaf longevity can result in a range of leaf traits limiting photosynthesis.

In our previous paper, we explored the growth, biomass allocation and photosynthesis of two Finnish birch provenances (northern 67°N and southern 61°N) in uniform conditions from the perspective of them exhibiting separate strategies by latitude (Tenkanen et al. 2020*b*). The results pointed to a northern syndrome of traits, which we tentatively call the ‘polar day syndrome’. In the northern provenance, we found a strong tendency toward higher relative allocation to roots (higher root mass fraction, lower shoot:root ratio) and higher *A*_net_ and *g*_s_ in several measurement temperatures, but lower leaf mass fractionation with fewer leaves and lower total leaf dry weight (DW) compared with the southern provenance (Tenkanen et al. 2020*b*). Northern tree provenances in general can reach higher levels of leaf area-based gas exchange (*g*_s_ and *A*_net_) than southern ones (Wang et al. 1998, Gornall and Guy 2007, Soolanayakanahally et al. 2009, 2015, McKown et al. 2014, Kaluthota et al. 2015, Tenkanen et al. 2020*a*). In the north, the photoperiod in principle allows for continuous photosynthesis, but benefitting from it will be limited by the short growing season. Therefore, in natural conditions, gas exchange measurements scaled up over the entire season could reveal converging carbon assimilation capacities in northern and southern provenances, but in an equally long growing season northern ones may have an advantage. Yet there are also notable differences in growth between northern and southern plants. At the end of the season, boreal trees interpret the lengthening night as a signal to cease growth. The more northern a provenance, the lower this critical night length requirement is (Viherä-Aarnio et al. 2006, Luquez et al. 2008) and in a common photoperiod northern provenances should cease growth earlier than southern ones. However, in a long enough common photoperiod, the differences in height growth between provenances from the north and the south become evident only after several growing seasons as the small yearly differences accumulate (Tenkanen et al. 2020*a*, 2020*b*). Despite the resulting reduced height growth due to the shorter growing season in the north, it is not yet conclusively known if northern and southern provenances have differences in growth rates during active growth. However, greater height growth rates have been observed in northern Scots pine (*Pinus sylvestris* L.) provenances than in southern ones, regardless of photoperiod (Oleksyn et al. 1992). In addition, root phenology may not be linked to above-ground phenology in the north and root growth may continue after shoot growth cessation (Blume-Werry et al. 2016, Radville et al. 2016).

Silver birch has a Europe-wide distribution range, with Finland covering the northern edge. Our focus is on the potential variation between provenances that in nature are neighboring, yet which inhabit areas with different photoperiods. In northern Finland, the sun does not set for an extended period during midsummer (24 h CL during the polar day which lasts ~ 1.5 months), while in the south of Finland the day includes a period of twilight or darkness. These photoperiods will remain unchanged even as the climate changes, posing different challenges for acclimation at different latitudes as tree species’ ranges shift northwards. In this new experimental setup we followed many of the same traits as in our previous paper (growth, biomass allocation, gas exchange) but with the addition of leaf senescence. We also used the same provenances (Tenkanen et al. 2020*b*) but subjected them to two different photoperiods. Our assumption was that traits related to phenology (height growth cessation, leaf longevity) and those related to biomass accumulation and fractionation are affected by the photoperiod. Here we refer to the 24 h continuous light treatment as CL and to the 19.5 h non-continuous light treatment as NCL. These treatments correspond to the growing season day-lengths at the provenances’ respective latitudes of origin, and the experiment lasted for the entire duration of a natural growing season (4 months). This setup allowed us to determine how the provenances respond to the length of their native and non-native photoperiod, and how strongly their photosynthetic and other leaf traits are under photoperiodic control in this extreme situation. We had the following hypotheses:

(i) In the NCL treatment, the northern provenance grows less than the southern one (due to early height growth cessation). Leaf longevity is lower in the northern provenance than in the southern one. However, the treatment modifies the leaf longevity of both provenances so that it is lower in CL than in NCL, comparable to the short lifespan of high-light leaves. Irrespective of provenance or treatment, plants with low leaf longevity have higher photosynthetic rates (especially *A*_mass_, but also *A*_net_) and higher stomatal conductance (*g*_s_) but lower LMA than plants with high leaf longevity.
(ii) Both provenances accumulate more total biomass in CL than in NCL. Regardless of treatment, the northern provenance has higher root mass fraction and lower leaf mass fraction than the southern one. In the northern provenance, the balance of relative biomass allocation between shoots and roots is different in NCL due to earlier shoot height growth cessation but continuing root growth, resulting in a lower root mass fraction in CL than in NCL.
(iii) Regardless of photoperiod, the northern provenance reaches higher levels of *A*_net_ than the southern one.

## Materials and methods

### Plant material and experimental setup

Genotypes of Finnish silver birch provenances were clonally reproduced from buds collected in 2009 and thereafter maintained in tissue culture. Northern genotypes (Ki7, Ki27) originate from Kittilä provenance (67°KI; 67°44′N, 24°50′E) and southern genotypes (Pu17, Pu18, Pu25, Pu30) from Punkaharju provenance (61°PU; 61°48′N, 29°19′E), both from natural forests situated far apart with different climatic conditions see Table 1 in both (Tenkanen et al. 2020*a* and Tenkanen et al. 2020*b*). In Kittilä and Punkaharju, the length of the growing season is 122.6 days and 161.8 days, mean air temperature over the entire year is −0.8 °C and 3.5 °C (the long-term average growing season temperature difference is 1.6 °C) and the number of snow cover days is 210.4 and 159.3, respectively.

**Table 1:** Model estimates and effect *P*-values for the provenances in the CL and NCL treatments. Statistically significant differences (*P <* 0.05) are in bold. DAP = days after potting, TLDW/TLNo = total leaf DW divided by total leaf number. Data, variables and units as in Figures 3–6 and Figures S2–S6 available as Supplementary data at *Tree Physiology* Online.

	CL		NCL		*P*-values		
Model	61°PU	67°KI	61°PU	67°KI	Provenance	Treatment	Interaction
Height – DAP	38.5	37.7	38.4	28.0	0.125	0.556	0.234
RGR – DAP	0.0115	0.0158	0.0106	0.0175	**0.045**	**0.019**	0.720
AGR – DAP	4.6	4.4	4.8	2.9	0.111	0.998	0.724
ΔLeaves – DAP	5.6	2.0	3.1	0.6	**0.022**	**0.030**	0.185
No. of leaves – DAP	30.7	21.5	26.1	14.1	**0.001**	**<0.001**	**<0.001**
ΔBranches – DAP	0.859	0.337	0.512	0.054	**<0.001**	**<0.001**	0.730
No. of branches – DAP	8.2	4.4	6.3	1.8	**<0.001**	**<0.001**	**<0.001**
							
Root DW	4.4	4.7	3.8	3.6	0.370	**<0.001**	**0.016**
Stem DW	5.6	4.1	4.4	3.2	0.201	**0.002**	0.328
Total leaf DW	4.7	3.5	3.9	1.7	0.323	**<0.001**	**0.031**
RMF	0.30	0.39	0.32	0.47	0.222	**<0.001**	**0.009**
SMF	0.38	0.28	0.37	0.33	**0.003**	0.066	**0.024**
LMF	0.31	0.27	0.32	0.18	0.314	**0.024**	**0.017**
Total plant DW	14.8	13.0	12.1	8.0	0.466	**<0.001**	**0.008**
Shoot:root ratio	2.39	1.66	2.14	1.32	0.162	**0.002**	0.496
TLDW/TLNo	0.0554	0.1030	0.0780	0.1255	0.162	**0.006**	0.986
							
Leaf longevity	38.2	44.6	42.0	53.3	0.281	0.101	0.389
Leaf area	19.0	16.9	26.3	18.7	0.696	0.492	0.062
Leaf DW	0.126	0.152	0.161	0.152	0.536	0.998	0.052
Stomatal density	16,390	10,191	13,968	9,178	**0.015**	0.287	0.256
Total number of stomata	326,284	205,807	360,749	240,272	0.263	0.136	NA
LMA	6.29	7.52	5.79	7.89	0.068	0.354	**0.045**
							
*A*_net_	3.6	5.5	5.4	6.3	**0.022**	0.186	0.070
*A*_mass_	0.534	0.773	0.859	0.571	0.155	**0.008**	**<0.001**
*g*_s_	0.0323	0.0620	0.0728	0.1030	**0.035**	**0.002**	0.971
WUE	114.9	97.3	82.7	70.0	0.168	**<0.001**	0.579

Micropropagated plants were grown for 3 weeks on rooting medium (1/2 MS medium with 0.5 mg l^−1^ indoleacetic acid in Phytagel). Rooted plants were moved to unfertilized peat:perlite 1:1 (v/v, sterilized at 121 °C for 60 min), randomly placed in seedling flats within propagator trays with transparent lids and pre-grown for 4 weeks in a growth chamber (Microclima MC1000, Snijders Labs, Tilburg, The Netherlands, with inner dimensions of height 123.5 cm, width 128.5 cm and depth 65 cm). Growth conditions at this stage were diurnal rhythm of 16/8 h light/dark including 1 h morning and evening gradients in illumination (14 h of full 100% light), constant day/night temperature of 22 °C and relative humidity (RH) of 60%. The diurnal rhythm tracked real-world time. Photosynthetically active radiation at the plant level was on average 150 μmol m^−2^ s^−1^ (LI-250 light meter, Li-Cor Inc., Lincoln NE, USA), which corresponds to the level of light the plants were kept at in vitro. About twice a week the plants were bottom watered with chamber-temperature DI water. At each watering, the trays were rotated around the chamber to randomize plant locations. Tray hoods were removed after 2 weeks to acclimatize plants to the RH prevailing in the chamber.

After pre-growth, plants were transplanted into 1.5 l pots on a mixture of unfertilized peat:vermiculite 3:1 (v/v, to a total weight of 300 g, sterilized at 121 °C for 60 min) with 2 g l^−1^ of lime. The potting day is denoted 0 DAP (days after potting). In total, 54 plants from four southern and two northern genotypes were divided into two treatments (2–6 plants/genotype/treatment), one chamber per treatment (27 plants/treatment, 10 plants of 67°KI and 17 plants of 61°PU per treatment). Six plants were later discarded, and the actual number of observations varied among measurements (e.g., due to damaged leaves; actual numbers indicated in figures). Equal numbers of plants from each genotype were randomly divided between the treatments. All plants within a genotype had an equal chance to receive either treatment and plant position within chambers was random. The treatments were (i) CL with a 24/0 h light/dark photoperiod and (ii) NCL with a 19.5/4.5 h light/dark photoperiod, to correspond to the midsummer (June–July) photoperiods at the latitudes of Kittilä and Punkaharju (Figure 1a). Irrespective of photoperiod, both treatments had a common 19.5/4.5 h thermoperiod of 20/16 °C temperatures. Night temperatures lower than this could result in early growth cessation for birch (Mäenpää 2012) and were thus not considered, and higher temperatures could synergistically increase CL-induced stress (Velez-Ramirez et al. 2011). Furthermore, a realistic thermoperiod may entrain some circadian rhythmicity, which might otherwise be lacking in the CL treatment (Velez-Ramirez et al. 2011). Relative humidity was constant at 60% for both treatments. Morning and evening were simulated with 1-h long gradients of light and/or temperature. In both treatments, light intensity was equal and varied between around 150–600 μmol m^−2^ s^−1^ from the bottom to the top of the highest plants and horizontally across the chambers during the experiment. Compared with full sun, the light intensity we used was low, which likely alleviated any possible CL-induced injury (Velez-Ramirez et al. 2011). Neither of the provenances showed visual symptoms of leaf chlorosis and necrosis that commonly occur as responses to CL (Velez-Ramirez et al. 2011) and high light levels (Ellsworth and Reich 1992) (for details on light intensity and quality, see Supplementary Methods S1 available as Supplementary data at *Tree Physiology* Online). At this stage, the plants were surface watered with equal volumes of 20 °C DI water with fertilizer (Pot Plant Superex, Kekkilä Finland, with NPK 16–4–24) about once a week. The first watering was with a 0.1% fertilizer solution (160, 40 and 240 p.p.m. of nitrogen, phosphorus and potassium, respectively) and later ones were with a 0.2% solution. This corresponds to about 19 kg N ha^−1^ year^−1^, a moderate fertilization level that has been used previously for silver birch (e.g., Mutikainen et al. 2000, Keski-Saari et al. 2005). At each watering, plants were randomized within and between chambers to minimize chamber effects. The first growth measurements were made at 4 DAP, at which point the plantlets were on average 1.4 cm high (ranging from 0.5 to 4 cm) and had on average 5.1 small leaves (range 2–9 leaves). As the experiment progressed, differences in height among plants and between treatments started to become more pronounced. Therefore, from 28 DAP until the end of the experiment the distances from the tip of the plants to the illumination were equalized by placing the pots on adjustable platforms.

**Figure 2.**
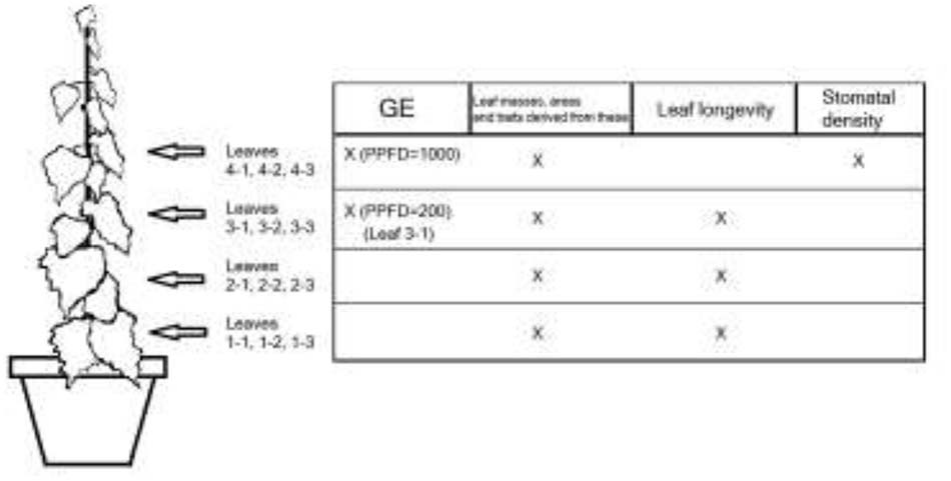
Schematic illustration of a silver birch shown with the groups of leaves marked during the experiment. Actual distances between the groups depended on the plant, but each group always consisted of three adjacent leaves. GE = gas exchange.

As the plants grew, we marked the youngest fully developed leaves of all plants, and two subsequent leaves above them (i.e., three vertically neighboring leaves). The youngest fully developed leaves were marked at 27, 41, 67 and 87 DAP and at the time of marking these were generally about the fourth to fifth leaf from apex. Thus, the three leaves of each group were about the same age among all the plants, while the age difference between groups was bigger. In most cases, some leaves were left unmarked between two vertically neighboring groups. Leaves were labeled with two numbers, the first indicating the group of three leaves (counted from the base) and the second the leaf position in the group (1–3, 1 being the lowest and oldest). This scheme enabled leaves of a relatively similar age to be followed in plants that differed in growth rates. Gas exchange measurements were mainly made on the first leaf of each group, while the rest were mainly reserved for determinations of leaf longevity, dry weights, leaf areas and stomatal density (Figure 2). If the correct leaves were not available, these were substituted with other leaves of the group.

All measurements were taken from the same spot on each leaf, from the right-hand side of the lamina when possible. All measurements were done either in the chambers the plants had been growing in or in a chamber with the same conditions.

### Growth, biomass and leaf traits

We followed the development of plant height and counted the total number of branches and leaves 15 times between 4 and 123 DAP. Height was measured to an accuracy of 0.5 cm. Relative height growth rate (RGR) was calculated as (1/ln(height))*(Δln(height)/ΔT), where Δln(height) is the difference in ln-transformed height since the last measurement and ΔT is the difference in time (days) since the previous measurement. Absolute height growth rate (AGR) was calculated as the height increment since the previous measurement. The number of leaves counted included only fully matured leaves. We examined both the change in the number of leaves and branches since the previous measurement (Δleaves and Δbranches) and the total number of leaves and branches. The observed Δbranches somewhat depended on the counter, resulting in response curves that may tend toward the negative even when that is not realistic (negative Δbranches would suggest breaking off of branches).

Leaves were sampled for mass and area twice. Leaves in group 4 were sampled at 113 DAP (1 day after the last gas exchange measurements) and the rest at 123 DAP during harvest. Leaf dry masses include the lamina and petiole. Leaf areas were measured from the lamina of detached leaves, excluding the petiole. Areas were determined in Fiji (Schindelin et al. 2012) from 600 d.p.i. scans of the adaxial side of flattened and dried leaves (without re-dampening). Images were first converted to 8-bit grayscale, then a threshold was applied to exclude the background and the lamina outlines discovered with Fiji’s particle analysis tools with the minimum pixel area size set to 50. Size calibration was done against an accurate caliper. Leaf mass per area was calculated as an average per individual plant by dividing leaf dry masses (lamina only) with dry leaf areas (lamina only). We present the results of leaf DW, leaf area and LMA combined from the two dates from pooled data of leaves of different age classes from different heights on the plant, giving an average of the plant (for details, see Supplementary Methods S1 available as Supplementary data at *Tree Physiology* Online).

Leaf longevity was monitored from all those marked leaves that were shed during the experiment, a total of 221 leaves from 38 plants. Plants were monitored at least once a week during 81–120 DAP, and leaves expected to abscise soon were monitored daily. Leaf longevity is expressed as a mean per plant of leaves abscised at different times during the experiment.

At 123 DAP, all leaves were harvested and weighed. Shoot diameter was measured with a caliper from a height about 1 cm above soil, and the shoot was cut from this location. Roots were frozen with the soil at −17**°**C, later unfrozen and washed clean of soil. All materials were dried at 70–80 °C for a minimum of 48 h (or until no change in mass) and weighed for dry mass. Root, shoot and leaf mass fractions (RMF, SMF, LMF) were calculated to depict the portion of each dry mass to plant total dry mass (g g^−1^). The shoot:root ratio was calculated from dry weights as (leaf total DW (g) + stem DW (g))/root DW (g). Total leaf DW divided by total leaf number (TLDW/TLNo) was calculated as an approximation of the average mass of an individual leaf.

For calculation of stomatal density, nail polish imprints of leaf abaxial surfaces were taken onto microscope slides from dry and pressed leaves of group 4. Imprints were peeled from the right-hand side of the lamina between the first and third leaf veins from the same spot where gas exchange had been measured. Five photographs were taken of each slide at 20× magnification from random locations (an area of 0.385 mm^2^ per photograph), avoiding leaf edges, broken areas and leaf veins. The stomata on each photograph were manually counted, excluding those touching the upper and right corners of the image, and an average was calculated from the five images. Stomatal density cm^−2^ was thereafter calculated, using a stage micrometer slide for size calibration. Total number of stomata was calculated by multiplying stomatal density with total leaf area (presented in the Supplementary Material available as Supplementary data at *Tree Physiology* Online).

### Gas exchange

Gas exchange was measured with the LI-6400 Portable Photosynthesis System OPEN Version 3.4.3 (Li-Cor, Inc., USA) from leaf 3–1 during 85–88 DAP (18–21 days after marking the leaf to allow leaf expansion and maturation). Measurements were divided to 4 days and were done between 08:00 and 16:00 h. Each plant was measured once during the morning (8:00–12:00 h) and once during the afternoon (12:00–16:00 h) to arrive at a daily average photosynthesis with two replicate measurements. The morning and afternoon measurements showed very similar distribution of values and could be pooled. All measurements were done while the plants were kept in prevailing chamber conditions (illumination and temperature), and the instrument was kept in chamber temperature throughout the experiment. As each individual plant was measured during 1 day only, the measurement order was randomized among and within measurement days—each individual from both treatments and all genotypes had an equal chance to be measured on any of the days and in any order, but the afternoon measurements were done in the same order as the morning measurements of that day. The airflow rate was 400 μmol s^−1^, reference cell [CO_2_] was 420 μmol mol^−1^, cooler block temperature was kept at 20 °C, sample cell RH was manually controlled to about 50–55% and photosynthetic photon flux density (PPFD) was 200 μmol photons m^−2^ s^−1^ (chamber ambient light intensity at the level of the measured leaves). Before each measurement was taken, the instrument was matched and leaves were acclimated in the measuring head for a minimum of 5 min or until values were stable (TotalCV% < 1%).

To examine whether measurements made at PPFD 200 μmol photons m^−2^ s^−1^ corresponded well enough to those made at light saturation, and to verify that our 4-day measuring campaign did not overtly affect the results, we also measured light-saturated day-time gas exchange once during 111–112 DAP between 11:00 and 15:00 h during maximal gas exchange (maximal *g*_s_). Measurements were made primarily from leaf 4–1 or if this leaf was not measurable (damaged, too small to be measured or missing) then from leaf 4–2 or leaf 4–3. Each individual from both treatments and all genotypes had an equal chance to be measured on either day and in any order. Measuring was done in the same way and with the same settings as previously, but PPFD was 1000 μmol photons m^−2^ s^−1^ to ensure light saturation. This PPFD is saturating, but not yet photoinhibitory for chamber-grown birch (Tenkanen et al. 2020*b*). The values of gas exchange (*A*_net_, *A*_mass_, *g*_s_, WUE) measured at a PPFD of 200 μmol photons m^−2^ s^−1^ corresponded well enough to those measured later at 1000 μmol photons m^−2^ s^−1^ (Figure S7 available as Supplementary data at *Tree Physiology* Online) to conclude that both measurements come from the same distribution and are valid. The former had more data points and smaller confidence intervals, and therefore the results presented here are based on those. However, we utilize the latter for correlation analyses.

Mass-based net photosynthesis, *A*_mass_ (μmol CO_2_ g^−1^ s^−1^), was calculated by dividing *A*_net_ with LMA, using the LMA measured at harvest. For this, we used the average LMA calculated from all available leaves of the plant at harvest.

### Statistical analyses

Data were analyzed with R version 3.6.3 (R Development Core Team 2020) using Rstudio version 1.2.5033 (RStudio Team 2019). Packages used included *nlme* (for mixed models) (Pinheiro et al. 2020), *lme4* (for generalized mixed models) (Bates et al. 2015), *car* (for tests on *lme4*-models) (Fox and Weisberg 2019), *emmeans* (for calculation of mean estimates, confidence intervals and post-hoc pairwise comparisons) (Lenth 2020) and *ggplot2* (for graphics) (Wickham 2016).

The linear region of height growth was modeled as a random intercept and slope model using Eq. (1):

(1) \begin{align*} y &= {\beta}_0+{\beta}_1\text{Prov}+{\beta}_2\text{Treat}+{\beta}_3\text{DAP}\nonumber \\ &+{\beta}_{123}{\text{Prov}}^{\,\ast\, }{\text{Treat}}^{\,\ast\,}\text{DAP}+ Gt+\varepsilon \end{align*}

where *y* = height (cm), *β*s = fixed effects, Prov, Treat and DAP = design matrices for provenance, treatment and DAP, DAP = time in days after potting, *Gt* = random effect of genotype, *ε* = error term. The slope and intercept of this model were tested by likelihood-ratio tests with the *emmeans* package.

Time series of RGR, AGR and Δleaves were modeled as random intercept polynomial mixed models, transforming the continuous predictor DAP to the *n*th power using Eq. (2):

(2) \begin{eqnarray*} y={\beta}_0+{\beta}_1\text{Prov}+{\beta}_2\text{Treat}+{\beta}_3{\text{DAP}}^{\text{n}}+\nonumber\\{\beta}_{123}{\text{Prov}}^{\,\ast\, }{\text{Treat}}^{\,\ast\, }{\text{DAP}}^{\text{n}}+ Gt+\varepsilon \end{eqnarray*}

where *y* = dependent variable, *n* = 2 for AGR and 3 for RGR and Δleaves. The time series of the number of leaves and the number of branches were modeled using the fixed and random structure of Eq. (2) and *n* = 3, but as Poisson regression. The time series of Δbranches was modeled using only the fixed structure of Eq. (2) and *n* = 3. The DAP was standardized before raising it to the third power for the models of Δleaves, Δbranches, number of leaves and number of branches to avoid convergence problems.

The models for leaf longevity, leaf area, leaf DW, stomatal density, LMA, root DW, stem DW, total leaf DW, mass fractions (RMF, SMF, LMF), total plant DW, shoot:root ratio, TLDW/TLNo and gas exchange (*A*_net_, *A*_mass_, *g*_s_ and WUE, both the dataset acquired with 200 and the set acquired with 1000 μmol photons m^−2^ s^−1^) were random intercept models using Eq. (3):

(3) \begin{equation*} y={\beta}_0+{\beta}_1\text{Prov}+{\beta}_2\text{Treat}+{\beta}_{12}{\text{Prov}}^{\ \ast\ }\text{Treat}+ Gt+\varepsilon \end{equation*}

The model for the total number of stomata was the same as Eq. (3), but without the interaction, due to convergence issues.

Residuals were visually diagnosed for homogeneity using plots of standardized residuals versus fitted values and for normality using Q-Q plots with standardized residuals. Heterogeneity was found in the residuals of about half of the models, but all models were nonetheless built to include a variance function of the same form for uniformity and simplicity (in none of the cases did the inference change whether a variance function was included or not). Whenever a model was modified, it was compared with the less modified model using the Akaike Information Criterion (AIC) (Burnham and Anderson 2002). In most models, the variance function improved the residuals as determined visually and by AIC, while in some models the residuals were only visually more homoscedastic. Models built with *lme4* (Poisson models) and the model for Δbranches did not include a variance function.

Before calculating regression coefficients and *P*-values of effects (provenance, treatment, interaction), the models were estimated by restricted maximum likelihood (REML), except for the Poisson models which were estimated with maximum likelihood (ML) using the Laplace approximation, and the simple linear model for Δbranches. For the Poisson models, ML-estimated full models were compared with ‘null models’ by likelihood ratio tests. However, these *P*-values tend to be anti-conservative, and the significant results should be interpreted with caution. Post-hoc *P*-values were calculated with Tukey’s HSD method. We do not present post-hoc *P*-values in figure captions or in text, due to different inference between them and main effect *P*-values.

Finally, a simple analysis of correlation coefficients was performed to look at linear relationships among some variables of interest. Due to few data points on different data levels, these were calculated by considering only the main (grand) correlation across treatments and provenances and excluding the genotype level, which means that we were mainly interested in correlations that were possibly strong enough to be present even among these confounding factors. For all correlations involving gas exchange data, we used the measurements made at PPFD = 1000 μmol photons m^−2^ s^−1^ (leaves of group 4, Figure 2), because we were able obtain stomatal density observations from these leaves. Correlations and full information on them are presented in Figure S8 available as Supplementary data at *Tree Physiology* Online.

We show our results as boxplots in Figures 4–6, but remind the reader that *n* < 15 in all instances of 67°KI in Figure 4, all instances of 67°KI in NCL and most stomatal density measurements in Figure 5 and all instances of 67°KI in NCL in Figure 6.

## Results

### Growth and biomass allocation

**Figure 3.**
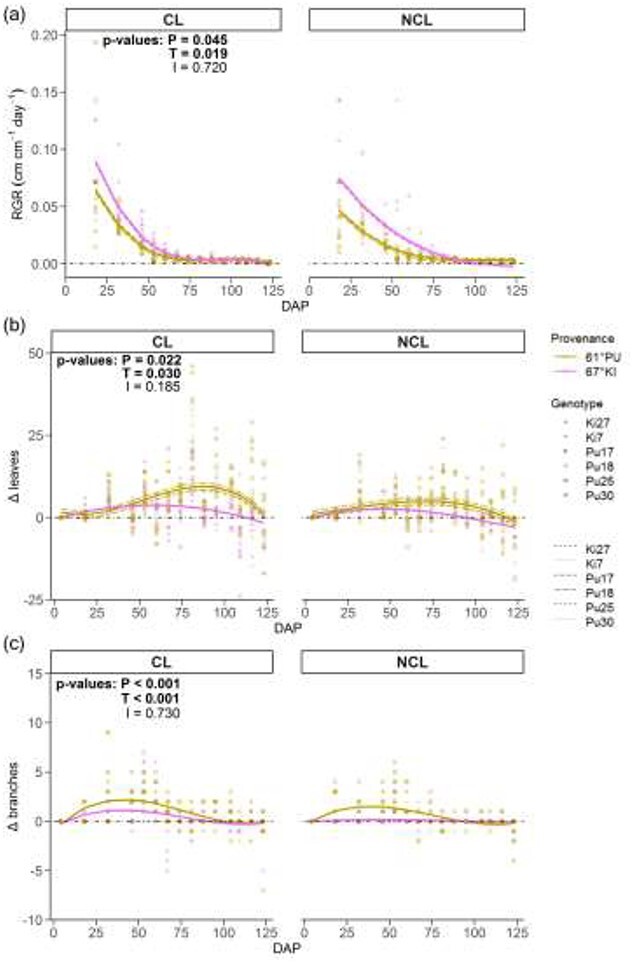
Time series of (a) RGR, (b) change in the number of leaves (Δleaves) and (c) change in the number of branches (Δbranches). CL = continuous light, NCL = non-continuous light. P, T and I = *P*-values for the provenance and treatment effect and their interaction, respectively. The genotype-level fits of 67°KI in (b) are almost identical to the provenance-level fits in both treatments and are therefore not shown. The genotype-level fits are missing from (c), as it is a simple fixed-effects model. 2–4 genotypes/provenance/treatment, *n* = 2–6 plants/genotype/treatment, 44–255 measurements/provenance/treatment.

After an initial lag phase, the height of all plants started to increase at around 32 DAP, along with an increase in branching and number of leaves (Figure S2b–d available as Supplementary data at *Tree Physiology* Online). The northern 67°KI had higher RGR than 61°PU in both treatments (Figure 3a, Table 1). However, toward the end of the NCL treatment the RGR of 67°KI decreased to zero and it became stunted compared with 61°PU (Figure 3a, Figures S1 and S2b available as Supplementary data at *Tree Physiology* Online). The difference in growth cessation is also visible in the curve for AGR (Figure S2a available as Supplementary data at *Tree Physiology* Online), although the main effects in AGR were non-significant (Table 1). For most of the 67°KI plants in NCL, height growth cessation appeared to have happened between around 80–95 DAP and the height of 67°KI clearly lagged behind 61°PU even before the growth cessation. In contrast, in the CL treatment, neither provenance showed signs of complete growth cessation by the end of the experiment. Further, as the experiment progressed, we had to discard five 67°KI plants from the NCL treatment because of failed growth, compared with having to discard only one plant from the CL treatment (one 61°PU plant). Both provenances had higher RGR in CL than in NCL (Figure 3a). The slopes of the model depicting the linear growth phase did not differ between provenances in either treatment and we did not find significant provenance or treatment effects in absolute heights during this time of active growth (Figure S2b available as Supplementary data at *Tree Physiology* Online). The intercept was 10.8 cm lower for 67°KI than 61°PU in NCL, but the intercepts did not differ in CL (Figure S2b available as Supplementary data at *Tree Physiology* Online).

In both treatments, 67°KI had a lower rate of leaf and branch formation, measured as the change in leaf or branch number (Δleaves, Δbranches) than 61°PU (Figure 3b and c, Table 1). Thus, there were less leaves and branches in 67°KI than in 61°PU (Figure S2c and d available as Supplementary data at *Tree Physiology* Online, Table 1). Both provenances had higher rates of leaf and branch formation (Figure 3b and c) and more total leaves and branches (Figure S2c and d available as Supplementary data at *Tree Physiology* Online) in CL than in NCL (Table 1). In addition, 67°KI started shedding leaves earlier than 61°PU in both treatments (Figure 3b), coinciding with height growth cessation. Significant interaction effects were found in the models of total amount of leaves and branches (Figure S2c and d available as Supplementary data at *Tree Physiology* Online). Post-hoc tests suggested that in the model for total leaf number the significant interaction arose from the difference between 67°KI and 61°PU in CL being non-significant (*P* = 0.205), while in the model for total branch number all post-hoc tests were significant (not shown) and no clear reason for the interaction was found.

**Figure 4.**
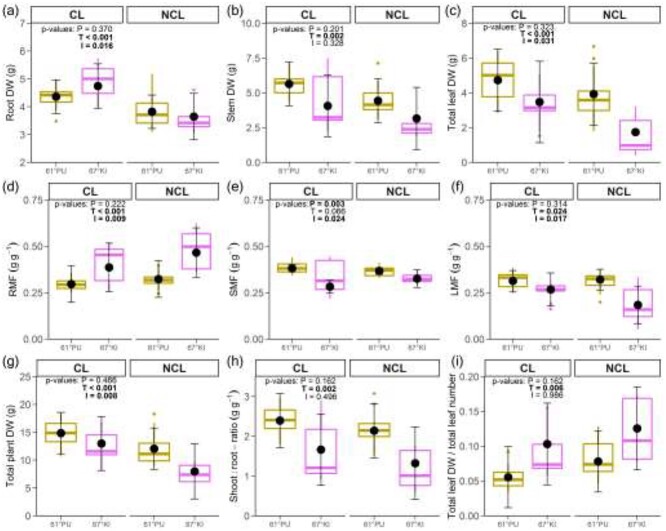
(a) Root dry weight, (b) stem dry weight, (c) total leaf dry weight, (d) root mass fraction, (e) shoot mass fraction, (f) leaf mass fraction, (g) total plant dry weight, (h) shoot:root ratio (shoot-part including leaves) and (i) total leaf dry weight divided by total leaf number. CL = continuous light, NCL = non-continuous light. P, T and I = *P*-values for the provenance and treatment effect and their interaction, respectively. Boxplots show distribution of raw data (whiskers denote largest/smallest observation less/greater than or equal to upper/lower hinge +1.5 * IQR), genotypes not shown for clarity. Black points and error bars are model estimated means and 95% confidence intervals. 2–4 genotypes/provenance/treatment, *n* = 2–6 plants/genotype/treatment, 5–17 measurements/provenance/treatment. Confidence intervals of 67°KI in the NCL-treatment in (c) have been omitted, because these were not in parameter space (negative lower bound, likely arising from large differences between genotypes, see Figure S3c available as Supplementary data at *Tree Physiology* Online).

The treatment affected many aspects of biomass accumulation, while biomass fractionation was less affected. Significant provenance differences were almost completely absent in the biomass data. Both provenances accumulated more root, stem and leaf biomass in CL than in NCL (Figure 4a–c, Table 1), leading to higher total plant DW for both provenances in CL than in NCL (Figure 4g). TLDW/TLNo, which was calculated as an approximation of the average leaf mass, was lower in CL than in NCL for both provenances (Figure 4i). Less biomass was partitioned to roots in CL, as root mass fraction was lower for both provenances in CL than in NCL (Figure 4d). Treatment did not affect allocation to stems; instead, post-hoc analyses showed that 67°KI in CL attained a lower shoot mass fraction than 61°PU in both CL and NCL (Figure 4e). Shoot:root ratio was higher for both provenances in CL than in NCL (Figure 4h). Leaf mass fraction had significant treatment and interaction effects (Figure 4f), but in post-hoc analyses there were no provenance or treatment differences.

### Leaf traits

**Figure 5.**
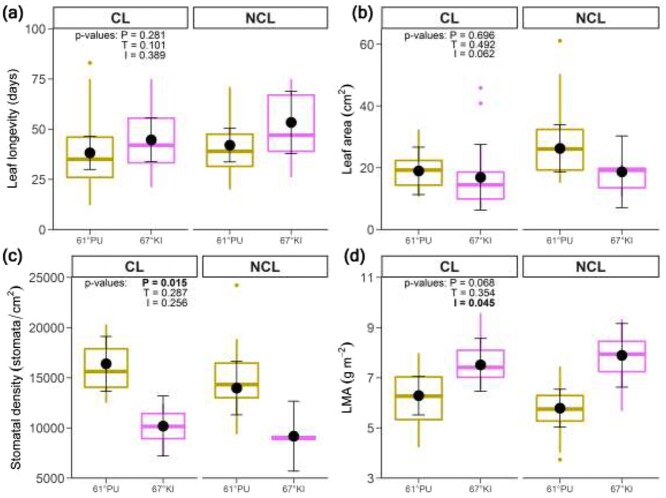
(a) Leaf longevity, (b) leaf area, (c) stomatal density and (d) leaf mass per area. Symbols as in Figures 3 and 4. 2–4 genotypes/provenance/treatment, *n* = 1–6 plants/genotype/treatment, 3–91 measurements/provenance/treatment. Genotype-level leaf trait data is available in Figure S4 available as Supplementary data at *Tree Physiology* Online.

Significant main effects were rare in the leaf trait data. Leaf longevity ranged between 12 and 83 days and averaged at 40.6 days if all leaves, all genotypes and both treatments are considered. There were no statistical differences between provenances or treatments in leaf longevity (Figure 5a, Table 1), leaf area (Figure 5b) or leaf DW (Figure S5a available as Supplementary data at *Tree Physiology* Online). Stomatal density was lower in 67°KI than in 61°PU (Figure 5c), but when the total number of stomata was calculated over the entire leaf area the significant provenance effect was not present (Figure S5b available as Supplementary data at *Tree Physiology* Online). There was a significant interaction term in the model for LMA (Figure 5d), and post-hoc comparisons showed that 61°PU had higher LMA in CL than in NCL.

### Gas exchange

**Figure 6.**
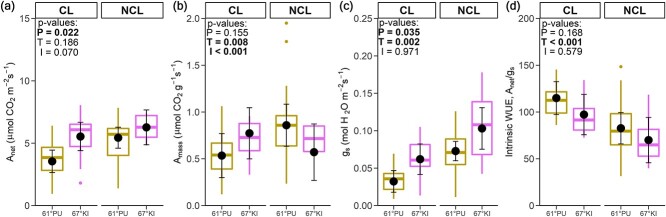
Gas exchange at PPFD = 200 μmol photons m^−2^ s^−1^. (a) Area-based net photosynthesis (*A*_net_), (b) mass-based net photosynthesis (*A*_mass_), (c) stomatal conductance (*g*_s_) and (d) intrinsic water-use efficiency (WUE). Symbols as in Figures 3 and 4. 2–4 genotypes/provenance/treatment, *n* = 2–6 plants/genotype/treatment, 10–34 measurements/provenance/treatment. The data are combined from 4 days of measurements, 2 measurements/plant/day (see text for details). The two outlier values of *A*_mass_ in 61°PU in NCL are not erroneous values—these belong to genotype Pu30 which exhibited both high *A*_net_ and *A*_mass_ (but also high spread in *A*_net_ and *A*_mass_) and low LMA in NCL. Genotype-level gas-exchange data are available in Figure S6 available as Supplementary data at *Tree Physiology* Online.

Area-based net photosynthetic rate (*A*_net_, Figure 6a) and area-based stomatal conductance (*g*_s_, Figure 6c) were higher in 67°KI than in 61°PU based on main-effect *P*-values (Table 1). However, water-use efficiency (WUE, *A*_net_/*g*_s_, Figure 6d) did not show provenance differences. When expressed on a leaf mass basis, net photosynthetic rate (*A*_mass_, Figure 6b) did not display provenance differences. Whereas *A*_net_ did not display a significant treatment effect, *g*_s_ was lower and WUE was higher in CL than in NCL (Figure 6c and d). *A*_mass_ had significant treatment and interaction effects.

Regardless of the significance or non-significance of the main effects, post-hoc tests revealed further details. Post-hoc tests did not find significant provenance differences in *A*_net_ or *g*_s_ within treatments, but if compared between treatments both were higher for 67°KI in NCL than for 61°PU in CL. Further, 61°PU had lower *A*_net_ in CL than in NCL, and both provenances had lower *g*_s_ and higher WUE in CL than in NCL. *A*_mass_ was higher for 67°KI and lower for 61°PU in CL than in NCL, explaining both the significant interaction and treatment main effects.

### Correlations

We tested the grand correlation across treatments and provenances for some variables for which we expected a significant relationship could plausibly endure this type of stacking of data despite confounding effects. We found significant correlations between LMA and *A*_mass_ (*r* = −0.40), LMA and *g*_s_ (*r* = 0.43), LMA and number of leaves (*ρ* = −0.48), *g*_s_ and stomatal density (*r* = −0.58) and *g*_s_ and total number of stomata (*r* = −0.53) (Figure S8 available as Supplementary data at *Tree Physiology* Online). We did not find correlations between leaf longevity and *A*_net_, leaf longevity and *A*_mass_, leaf longevity and *g*_s_, leaf longevity and LMA, or LMA and *A*_net_ (Figure S8 available as Supplementary data at *Tree Physiology* Online).

## Discussion

### Photoperiod affected growth cessation and allocation patterns, while leaf traits remained mostly unmodified

We propose the polar day trait syndrome of the northern provenance of silver birch in its natural habitat with CL to include good growth rate and good area-based gas exchange capacity. On the other hand, total carbon sequestration of northern birches should be limited by the short growing season and low leaf biomass. These traits are susceptible to modification by the photoperiod, as the treatment affected gas exchange, growth and biomass of both provenances. A range shift from areas of NCL toward areas of CL should therefore affect photosynthetic rate and biomass accumulation, but would expose southern plants to the risks of unsynchronized phenology (in the current climate).

The photoperiod dramatically affected the height growth of the northern 67°KI, while the southern 61°PU was mostly unaffected. Although RGR was clearly higher in 67°KI than 61°PU in both treatments, in the NCL treatment 67°KI stopped height growth earlier than 61°PU and therefore attained shorter final height. This was expected, as northern birches have a shorter requirement for critical night length to induce height growth cessation (Viherä-Aarnio et al. 2006, Luquez et al. 2008). Previously, we have similarly observed 67°KI to produce terminal buds after around 3 months, when grown in a 20/4 h photoperiod (Tenkanen et al. 2020*b*), which is half an hour longer than the NCL treatment used here. Further, in CL the regression line of the model depicting the linear growth phase of 67°KI crossed that of 61°PU and in AGR the curve of 67°KI surpassed that of 61°PU at the midpoint of growth, matching the time when the regressions crossed. Our results point to higher height growth rates in 67°KI compared with 61°PU in long photoperiods, similar to higher height growth rates in northern Scots pine populations than in southern ones (Oleksyn et al. 1992).

We found no support for the hypothesis that leaf longevity would be lower in 67°KI than in 61°PU. For populations of temperate to boreal evergreens, needle longevity is usually higher in northern populations than in more southern ones (Reich et al. 1996, Pensa et al. 2007), but with temperature rather than latitude being a strong determinant for needle retention (Jalkanen et al. 1995). However, evergreen needles operate under a different time scale than deciduous leaves, making comparisons difficult. For deciduous species, leaf longevity usually declines toward cooler temperatures (toward north) in species-wide comparisons (Kikuzawa et al. 2013). Even if such global correlations may be misleading, if the aim is a within-species comparison, leaf longevity seems to be quite plastic to the growth environment (Reich et al. 1996). In the north, there appears to be considerable pressure toward increasing LMA (Reich et al. 1996, Poorter et al. 2009, Kaluthota et al. 2015, Jankowski et al. 2019), which could also extend the leaves’ payback time (Williams et al. 1989, Poorter et al. 2006) and leaf longevity. Counterevidence has also been presented, where LMA decreases toward lower mean annual temperature (Tullus et al. 2021), which could have different implications for leaf construction cost and longevity. Nevertheless, LMA and leaf longevity are not always clearly positively correlated in deciduous plants (Poorter et al. 2009), and in the current experiment LMA and leaf longevity did not correlate. We also hypothesized correlations between leaf longevity and gas exchange rates, but did not find any. Further, we did not find evidence that leaf longevity would have been significantly affected by photoperiod. Generally, high-light leaves should have faster leaf turnover but be suited for favorable conditions, while low-light leaves should take longer to become contributing net sources instead of sinks to ‘pay back’ the photosynthate lost in the construction of the leaves (Williams et al. 1989, Poorter et al. 2006). It is likely that our chamber light levels were not high enough to cause overt stress or a decrease in leaf longevity, contributing to the non-significant treatment effect (for an extended discussion on possible light-mediated effects on phenology, see Supplementary Methods S1 available as Supplementary data at *Tree Physiology* Online).

Johnsen and Seiler (1996) observed that northern black spruce (*Picea mariana* Mill.) reacted to an extended photoperiod by increasing total biomass, shoot:root ratio and RGR (although it should be noted that their northernmost provenance did not originate from above the Arctic circle and their extended photoperiod was not CL). We also saw higher total biomass, shoot:root ratios and RGR in both provenances in the CL treatment than in the NCL treatment. Most plants increased their root, stem and total leaf DW, and therefore increased total plant DW in CL. Although the allocation (fractionation) patterns clearly changed only in the roots in response to photoperiod, with smaller relative biomass allocation to roots in CL than in NCL, the mass fractions (RMF, SMF, LMF) of 67°KI reacted more to the treatment than the mass fractions of 61°PU. These effects on mass, mass fractions and shoot:root ratios can arise from differences in growth cessation, which was delayed in CL (and which had a bigger effect on 67°KI), because CL does not accumulate the critical night length signal and therefore exerts an extended photoperiod on the provenances. In gymnosperms, shoot growth cessation shifts the balance of biomass accumulation from shoots to roots (Oleksyn et al. 1992, Oleksyn et al. 1998, Lahti et al. 2005). In addition, since evergreens may continue to accumulate carbohydrates around the year, peak root growth is not necessarily linked to peak leaf growth (Radville et al. 2016). There is also more general evidence across arctic plant communities that above- and below-ground phenology may be unlinked in northern ecosystems where high root biomass is favored (Blume-Werry et al. 2016, Radville et al. 2016), implying that root growth can continue after shoot growth cessation. Nevertheless, it should be noted that the shoot:root ratio after one growing season is still susceptible to phenotypic adjustment later in time, as woody plants may in general have a tendency to decrease their shoot:root ratio during their first years of growth and increase it later (Ledig et al. 1970).

In the CL treatment, 61°PU increased its stem and root DW while decreasing its RMF and increasing its shoot:root ratio compared with its native photoperiod. Therefore, we were able to show that in response to a longer photoperiod, RMF can decrease (and shoot:root ratio can increase) even if absolute root mass increases. When grown in a photoperiod longer than its native seed origin, paper birch (*Betula papyrifera* Marsh.) gains height and above-ground biomass and biomass fraction, but loses root mass fraction and therefore has a higher shoot:root ratio (Tedla et al. 2019). However, Tedla et al. (2019) did not find a significant difference in root DW. They further discuss the counterintuitive nature of such an apparent decrease in below-ground allocation, as it should logically rather increase with increasing allocation to the shoot (to keep up with the increased water and nutrient demand), and conclude that the lower RMF in the longer photoperiods mainly reflect the higher above-ground SMF (Tedla et al. 2019). Our results may negate this apparent paradox and instead indicate an overall sturdier plant with increased carbon uptake in the long photoperiod.

### Continuous light downregulated photosynthesis and increased sink sizes, but provenance differences in gas exchange persisted irrespective of photoperiod

Photosynthetic capacity is often lower in CL, because the accumulation of sugar and starch downregulates photosynthesis and potentially triggers leaf senescence (Velez-Ramirez et al. 2011). We measured gas exchange toward the end of the growth season, which enabled the plants to acclimate to CL. Thus, it is possible that some downregulation of carbon assimilation happened in response to sugar accumulation while simultaneously, downstream sink sizes were being increased. Our main effect and post-hoc tests for gas exchange disagreed—this is possible as the tests answer different questions. Stomatal conductance (*g*_s_) was unambiguously lower and WUE was higher in CL than in NCL, but there was no conclusive treatment effect in *A*_net_. The results of *A*_mass_ are explained by the significant treatment and interaction effects. Thus, CL increased *A*_mass_ in 67°KI and decreased it in 61°PU. However, this result is tentative because the *A*_mass_ data of 67°KI in NCL was very small. Both *A*_net_ and *g*_s_ had significant provenance main effects without significant interactions, despite the ambiguity between main effect and post-hoc tests. Therefore, we conclude that *A*_net_ and *g*_s_ were higher in 67°KI than in 61°PU, which was also seen in our previous chamber experiment (Tenkanen et al. 2020*b*). This agrees with the literature on higher gas exchange rates in northern provenances compared with southern ones (Wang et al. 1998, Gornall and Guy 2007, Soolanayakanahally et al. 2009, 2015, McKown et al. 2014, Kaluthota et al. 2015, Tenkanen et al. 2020*a*). Notably, however, a provenance difference was not found when photosynthetic rate was expressed on a mass basis (*A*_mass_). Although *g*_s_ correlated with stomatal density, 67°KI had lower stomatal density than 61°PU, and therefore in future comparisons of gas exchange between provenances it is advisable to also measure stomatal aperture and stomatal guard cell size.

We were able to affirm our hypothesis that 67°KI attains a lower root mass fraction in CL than in NCL, possibly due to earlier growth cessation disturbing its biomass allocation (Oleksyn et al. 1992, Oleksyn et al. 1998, Lahti et al. 2005). This was further evidenced by the higher shoot:root ratio of 67°KI in CL than in NCL. We could not replicate our previous results of a significantly higher root mass fraction or a lower leaf mass fraction in 67°KI than in 61°PU (Tenkanen et al. 2020*b*), as hypothesized, but in both treatments 67°KI had noticeably fewer leaves than 61°PU. Differences in branching and leafing may be intrinsic growth strategies between northern and southern birches. Continuous light increases available resources but also represents continuous stress for a plant—excess excitation energy will produce oxidizing reactive oxygen species (ROS) unless the plant can increase the size of its sinks, and indeed CL seemed to strengthen all sinks (root, stem and total plant DW). Both provenances also produced more leaves in CL than in NCL.

On the whole, we did not observe obvious signs of CL-induced injury in either provenance despite prolonged growth in CL, but instead observed increasing sink sizes and photosynthetic downregulation. Nevertheless, compared with the photoperiod both provenances were kept at during in vitro growth (16/8 h), it is presumable that CL (24/0 h) did cause at least some light-induced stress to photosystems. It is possible that the stress was worse for 61°PU, but based on the current experiment this cannot be directly known. In addition, the possible differences between provenances in their capacity for the repair and upkeep of photosystems or ROS scavenging are unknown. In mountain birch (*Betula pubescens* ssp. *czerepanovii*), the xanthophyll cycle is able to maintain periodicity during the CL of the Arctic summer, which means that the cycle is able to relax (re-epoxidize) during the ‘nights’, in contrast to Scots pine and Alpine bearberry (*Arctostaphylos alpina* (L.) Nied.), both of which remain in energy-dissipating states (Fernández-Marín et al. 2018). This phenomenon suggests that these birches may have had time to adapt parts of their photosynthetic machinery to CL after post-glacial recolonization, and this may hold true for the rapidly establishing silver birch as well.

We suggest that further evidence of northern birches exhibiting a ‘polar day syndrome’ is discoverable, including preferable investment in a small amount of robust, high-efficiency leaves with possibly relatively high gas exchange rates. These should be precisely quantified by looking at the connections between traits that are central for light interception, photosynthesis and leaf longevity between northern and southern trees in different natural conditions. Leaf longevity is a highly plastic trait, which is likely to adapt to the changing climate. It would be of interest to more exactly elucidate how much of the variation in leaf age is due to the provenance (location of origin), and how much this can be modified by changing the photoperiod. This question should be studied in conjunction with functional traits such as LMA and photosynthesis, while taking into account the potential for, or lack of, acclimation in all these traits. In addition, the high RGR of 67°KI would suggest that the height growth of these northern trees is mainly limited by the environment, and in NCL by their earlier growth cessation. We would like to see a study where the RGR of northern provenances would be investigated with a much larger data set in different photoperiods.

## Conclusions

The photoperiod in our experiment most noticeably affected height growth cessation, patterns of allocation and stomatal conductance. The northern 67°KI provenance had early height growth cessation in NCL and thus, compared with the southern 61°PU provenance, had less time to accumulate and distribute biomass. Otherwise, the direction of change in most traits was similar for both provenances in response to the photoperiod, with both provenances responding to CL by downregulating photosynthesis, increasing RGR and building-up sink sizes throughout the plant, while leaf longevity and leaf functional structure were not notably affected. While CL at a moderate irradiance did not cause visible damage to either provenance, resistance to CL-induced damage would also seem advantageous in the north, and the capacity for this may differ between northern and southern provenances. Unraveling these possible ecotype differences in damage prevention and repair systems will be important for predicting possible future range shifts. With the Arctic warming, silver birch populations with currently southern distributions may find suitable habitats further north. Studies using reciprocal photoperiods can elucidate the effects of such migrations, while also simulating the transfer of provenances from the north to the south, which is useful for planning forestry plantings and selection of seed origin.

## Supplementary Material

Tenkanen_et_al_manuscript_Tree_Physiology_supporting_information_tpac104
