# Correction to: Polar day syndrome: differences in growth, photosynthetic traits and sink-size patterns between northern and southern Finnish silver birch (*Betula pendula* Roth) provenances in native and non-native photoperiods

**DOI:** 10.1093/treephys/tpad003

**Published:** 2023-01-23

This is a correction to: Antti Tenkanen, Markku Keinänen, Elina Oksanen, Sarita Keski-Saari, Sari Kontunen-Soppela, Polar day syndrome: differences in growth, photosynthetic traits and sink-size patterns between northern and southern Finnish silver birch (Betula pendula Roth) provenances in native and non-native photoperiods, *Tree Physiology*, Volume 43, Issue 1, January 2023, Pages 16–30, https://doi.org/10.1093/treephys/tpac104

In the originally published online version of this manuscript, there was an error in Figure 6: the subplots labelled ``CL'' and ``SD'' were incorrect and should be ``CL'' and ``NCL'' respectively.

**Figure 6:**
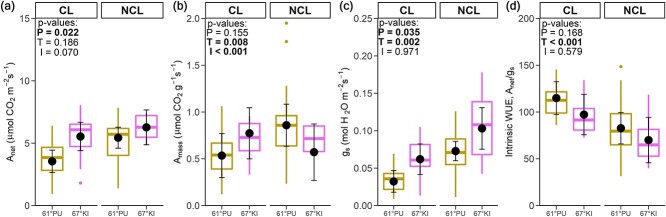


This error has now been corrected.

