# Supplementary material for: Polar day syndrome: differences in growth, photosynthetic traits and sink-size patterns between northern and southern Finnish silver birch (Betula pendula Roth) provenances in native and non-native photoperiods
Source: Tree Physiol. 2022 Sep 1;43(1):16–30. doi: 10.1093/treephys/tpac104 (PMC9833867; doi:10.1093/treephys/tpac104)
Supplement: Tenkanen_et_al_manuscript_Tree_Physiology_supporting_information_tpac104 [file tenkanen_et_al_manuscript_tree_physiology_supporting_information_tpac104.docx]

## Tree Physiology Supporting Information

Article title: **Polar day syndrome: Differences in growth, photosynthetic traits and sink-size patterns between northern and southern Finnish silver birch (*Betula pendula* Roth) provenances in native and non-native photoperiods.**

Authors:

*Antti Tenkanen, Markku Keinänen, Elina Oksanen, Sarita Keski-Saari* &

*Sari Kontunen-Soppela*

**The following Supporting Information is available for this article:**

**Supplementary Methods S1:** Extended context for the experimental setup

**Supplementary Figure S1:** Photographs of plants

**Supplementary Figure S2:** Time-series of AGR, height, total number of leaves and total number of branches

**Supplementary Figure S3:** Genotype-level biomass-related data

**Supplementary Figure S4:** Genotype-level leaf trait data

**Supplementary Figure S5:** Leaf dry weight and total number of stomata and their genotype-level data

**Supplementary Figure S6:** Genotype-level gas exchange data

**Supplementary Figure S7:** Gas exchange data at PPFD = 1000 µmol photons m^-2^ s^-1^

**Supplementary Figure S8:** Linear correlations

**Supplementary Methods S1:** Extended context for the experimental setup

At the latitude of the northern provenance (Kittilä, 67°44’N), the polar day lasts for about 1.5 months. Afterwards, the day-length shortens rapidly. Less than three weeks before the beginning and three weeks after the end of the polar day, the day-length at this latitude is around 19.5h, which already corresponds to the non-continuous light (NCL) treatment in this experiment and quite accurately to the day-length at the latitude of the southern provenance (Punkaharju, 61°48'N) at the midpoint of its growing season. Therefore, in our 4-month experiment, plants received continuous light (CL) for a longer period than in nature at 67°N. To study the developmental effects of CL in growth chambers on plants from different latitudes, a choice has to be made between giving the plants in the CL and NCL treatments either differing intensities (dosages) of light to reach a daily average equal dose, or equal intensities resulting in the CL-treatment receiving more light overall. The former may not be practical, as growth chamber illumination is often of relatively low power to begin with, resulting in poor growth if it is further diminished. In this study the latter was chosen, thus also eliminating differences in peak irradiance. Therefore, it is possible that CL in our experiment could affect leaf longevity and other traits in a manner similar to high light through increasing the total absorbed photosynthetic photon flux density (PPFD). However, these effects could also interact with the signaling effect of CL on plant diurnal rhythms. Additionally, in nature, the irradiance and spectrum of light has daily variation, which is dependent on latitude (because in the north the sun is always at a lower elevation angle). Even in the Arctic, although the sun never sets during the growing season and light intensity thus never reaches zero, light is not constant between “day-” and “night-time”. Indeed, at midnight, irradiance can reach very low levels, depending on cloud-cover (Velez-Ramirez et al. 2011, Fernández-Marín et al. 2018). Therefore, it is likely that plants can still use CL at high latitudes as a part of their diurnal signaling. The CL-treatment in this paper can be considered as both an extended photoperiod and an extended growing season, but we did not incorporate daily variation in light quantity and quality into our setup.

Solar elevation and thereby the quantity and spectral quality of light vary depending on latitude, time of year and time of day. Yearly means of summed daily irradiances are 12239.6 W/m^2^ for Kittilä and 14007.8 W/m^2^ for Punkaharju (difference 1768.1 W/m^2^), while the same means for the growing season (depicted in main text Fig. 1b) are 23070.0 W/m^2^ for Kittilä and 22733.2 W/m^2^ for Punkaharju (difference 336.8 W/m^2^ – note that this difference heavily depends on the definition of the growing season, and that although in this case Kittilä has a higher mean, all individual daily means are higher for Punkaharju). The highest solar elevations during the growing season are 45.9 degrees at Kittilä (temporally coinciding with CL) and 51.9 degrees at Punkaharju. Solar angle affects the ratios of blue to green (B:G), blue to red (B:R) and red to far-red (R:FR) radiation, and the atmospheric composition modifies the spectrum more as the sun elevation angle decreases (Pecot et al. 2005, Kotilainen et al. 2020). B:G, B:R and R:FR are sensed by plants through photoreceptors sensitive to blue, red and far-red, namely cryptochromes and phytochromes. These mediate a wide array of functions such as shade or neighbor avoidance and affect growth responses, including leaf expansion and thereby LMA (Pecot et al. 2005, Sellaro et al. 2010, Kalaitzoglou et al. 2019). Furthermore, in the field, plants are exposed to changes in day-length and thereby to changes in the R:FR spectral ratio. R:FR is reduced during low sun elevation angle (Chiang et al. 2019, Kotilainen et al. 2020), and during the growing season in northern Finland the sun is above the horizon but the elevation angle is low for a considerable part of the day. The signaling element of the change in light quality during morning and evening is thus affected throughout the season, and this may be important for plants at higher latitudes (Chiang et al. 2019). Changes in R:FR can affect growth (Tsegay et al. 2005), and phenology (Brelsford et al. 2019), but the exact interactive effects between light quality and quantity are unclear (Chiang et al. 2019). We did not modify chamber light quality as the experiment progressed, and it can therefore be asked whether this could have affected the phenological results dependent on light-signaling (height growth cessation, leaf longevity). It is highly unlikely that the height growth cessation results would have been decisively different, because it is under strong photoperiodic control (Tenkanen et al. 2020a and references therein). Leaf longevity, on the other hand, is a highly plastic trait, heavily modified by several factors such as temperature (Reich et al. 1996, Kikuzawa et al. 2013). A decreased R:FR ratio can reduce leaf longevity (Rousseaux et al. 1996), and thereby, in a setup incorporating more realistic light quality, the leaf longevity of plants in the “northern” CL treatment could have shown lower leaf longevities than in the current setup.

It should be noted that based on this study, it is not possible to conclude that (northern) birches would thrive without any external diurnal signals, as the plants were given realistic thermoperiods (and realistic photoperiods during the pregrowth-phase). These may have entrained daily rhythmicity e.g. in the plants’ ROS-scavenging capacity, which then could have persisted in the CL-treatment even in the absence of external light signals (Velez-Ramirez et al. 2011).

Our leaf sampling scheme enabled us to determine whether the measurements had an effect on leaf traits due to stress. The data were mainly from leaves of the upper half of the plant and measurements did not have an obviously discernible effect on the distributions of leaf longevities, leaf dry weights, leaf areas, LMA or stomatal densities among leaves (although leaf longevities varied a lot within each genotype irrespective of leaf position and stomatal density was measured only from leaves of group 4). Therefore, to increase data size, in the main text we present these results from pooled data of leaves of different age-classes, giving a representative average of the plant. For leaf DW, leaf area and LMA we additionally pooled data from two days. These days were adjacent to each other (113 DAP and 123 DAP at harvest) and the distributions of values were not very different between the dates. Unpooled data of these traits is shown below in Supplementary Methods Figures S1 and S2.


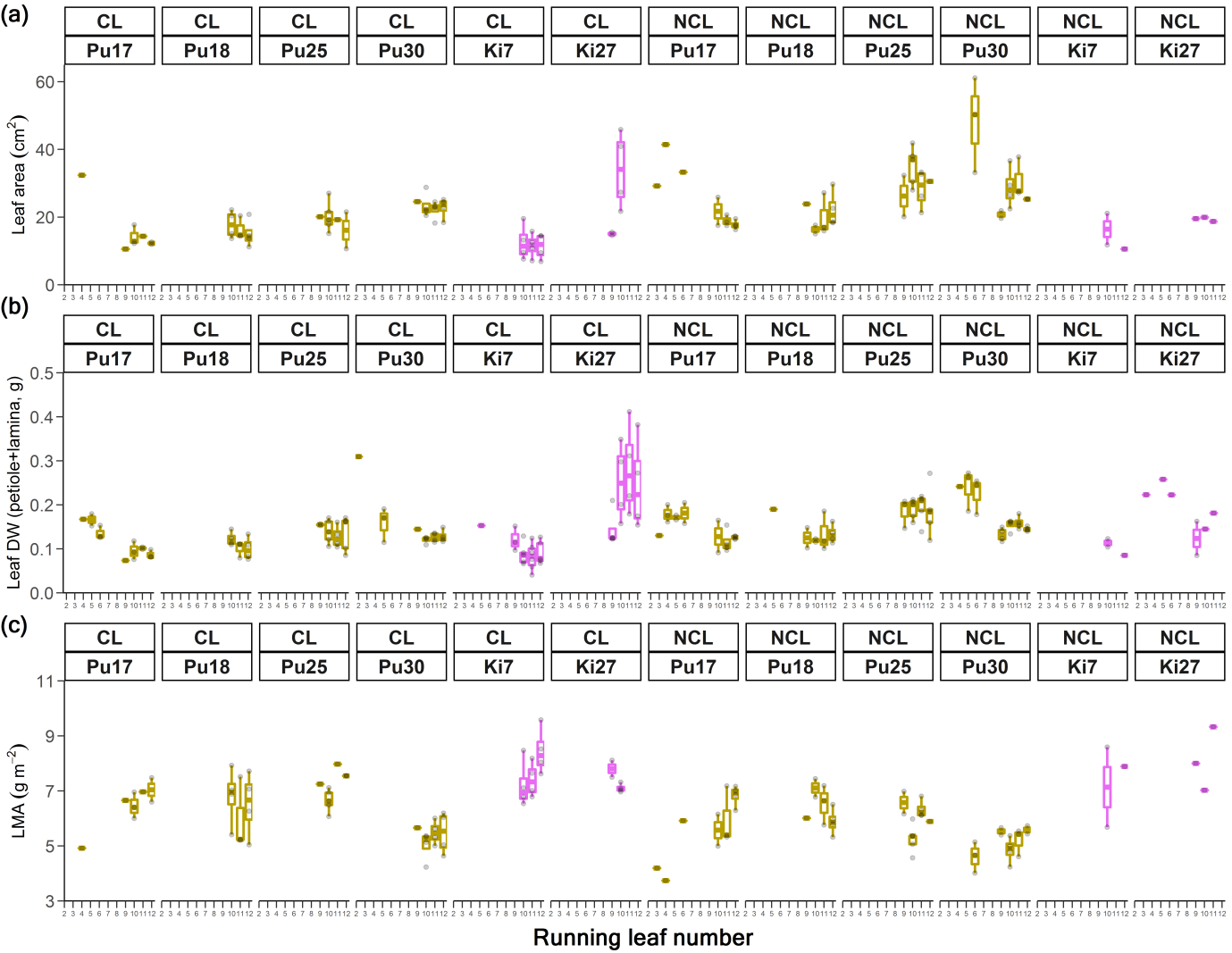


**Supplementary Methods Figure S1.** Distributions of values of each sampled leaf for comparison of leaves within each genotype in the continuous light (CL) treatment and in the non-continuous light (NCL) treatment for **(a)** leaf area, **(b)** leaf DW and **(c)** LMA. Leaves are numbered with a running number instead of the notation in the main text (number 1 corresponding to leaf 1-1, number 2 to 1-2 etc.). Data pooled from leaves sampled at 113 DAP and 123 DAP (harvest). Leaf longevity and stomatal density not presented.


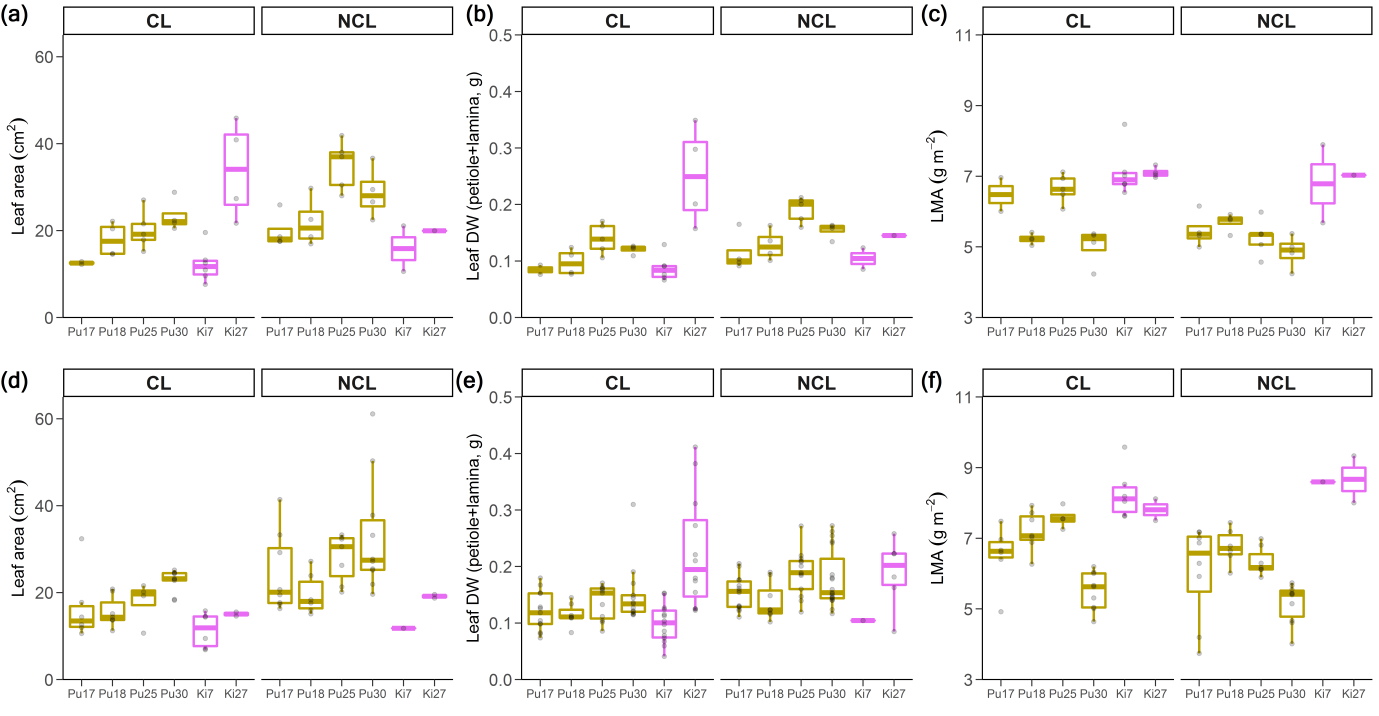


**Supplementary Methods Figure S2.** Distributions for comparison of sampling dates. Values of leaves sampled at 113 DAP for **(a)** leaf area, **(b)** leaf DW and **(c)** LMA and of leaves sampled at 123 DAP (harvest) for **(d)** leaf area, **(e)** leaf DW and **(f)** LMA in the continuous light (CL) treatment and in the non-continuous light (NCL) treatment. Data pooled from these is presented in the main text Figure 5, Supplementary Figure S5 and in the Supplementary Methods Figure S1.

**(a) Continuous light (CL) (b) Non-continuous light (NCL)
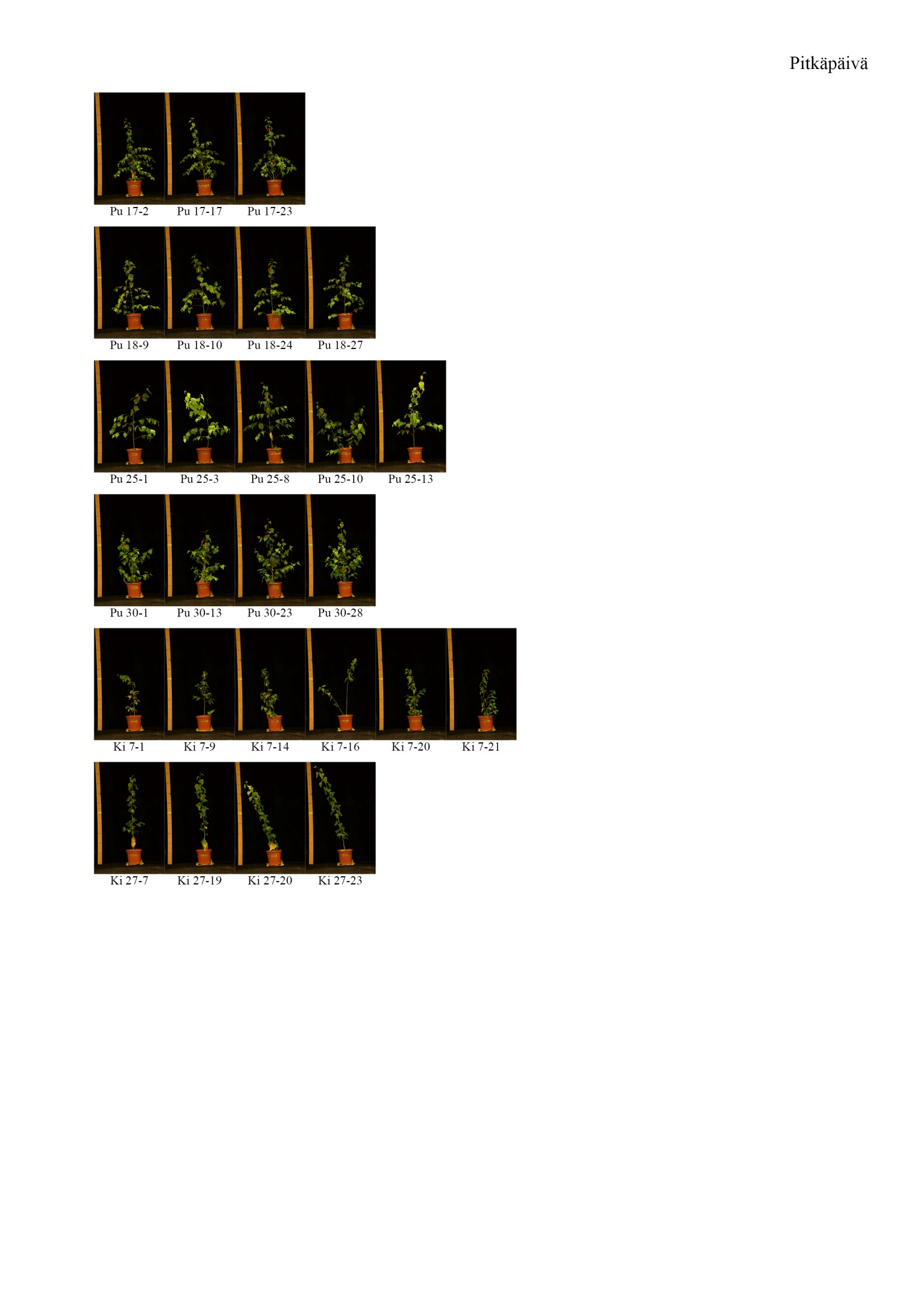

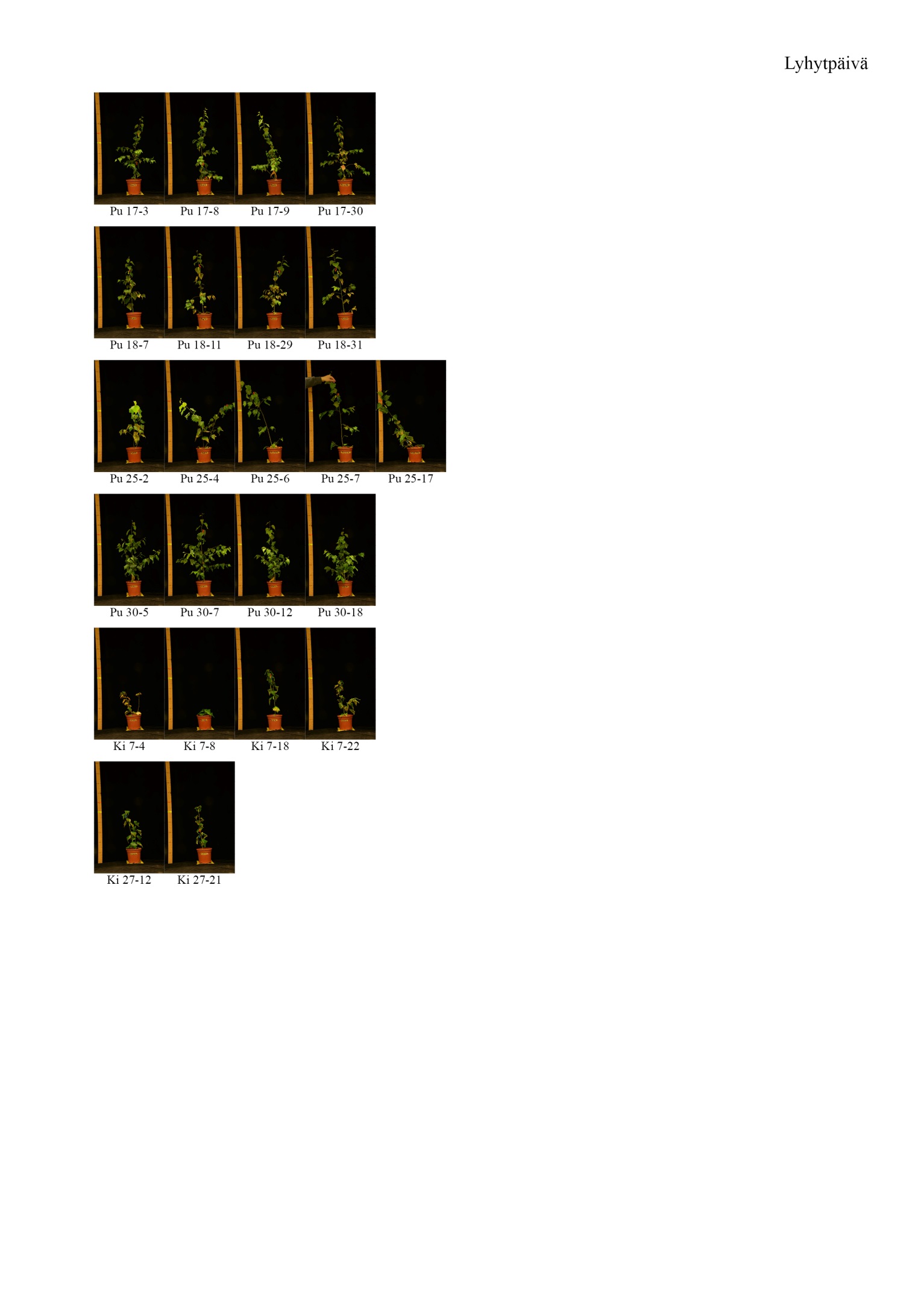
**

**Supplementary Figure S1.** Photographs of plants in the **(a)** continuous light (CL) treatment and in the **(b)** non-continuous light (NCL) treatment photographed at 111 DAP nearing the end of the experiment, sorted by genotype. By the time of photographing, one plant from the CL-treatment (Pu17-6) and four plants from the NCL treatment (Ki7-3, Ki7-10, Ki27-1 and Ki27-2) had been discarded (due to failed growth) from the originally potted 54 plants. Plant numbering here is related only to initial randomization.


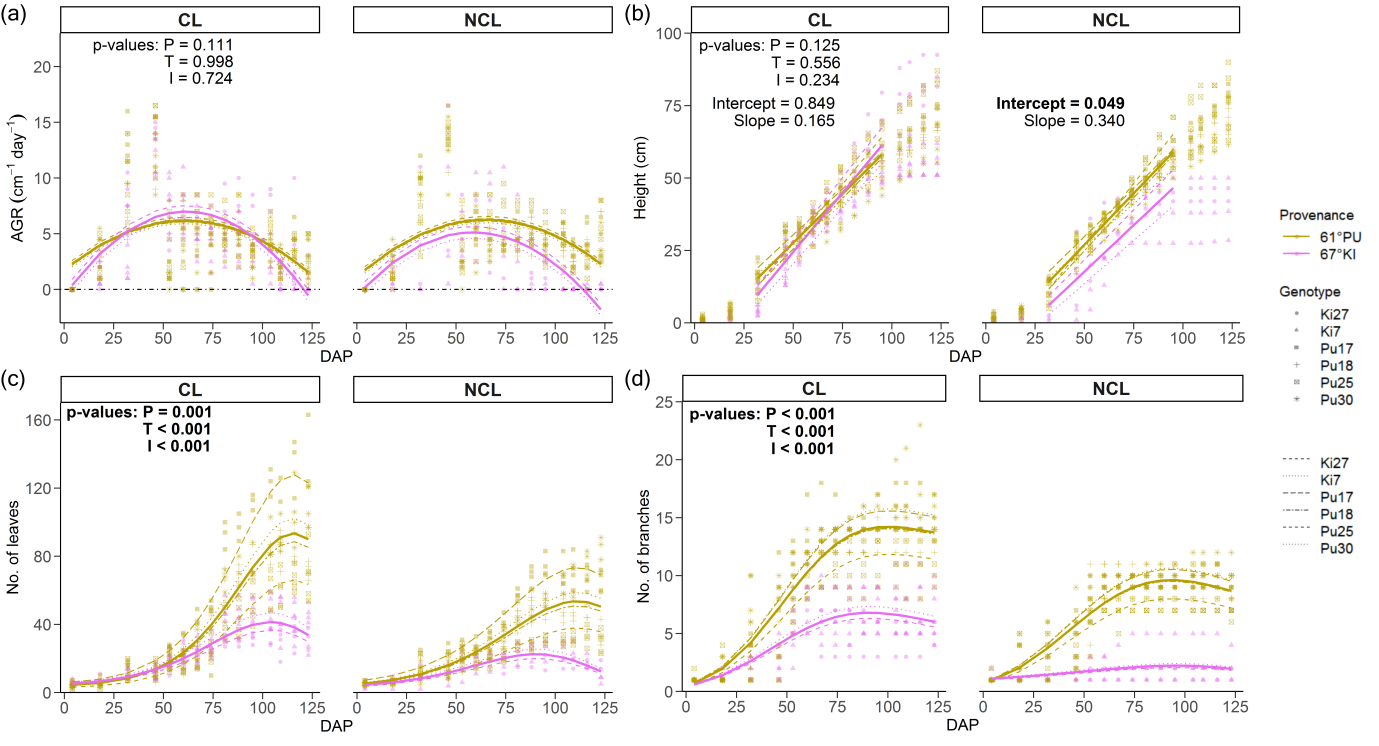


**Supplementary Figure S2.** **(a)** Absolute height growth rate, **(b)** height, **(c)** total number of leaves and **(d)** total number of branches of the 61°PU and 67°KI provenances. CL = continuous light, NCL = non-continuous light. P, T and I = *p*-values for the provenance and treatment effect and their interaction, respectively. For height, the *p*-values of the intercept and slope of the linear models are shown as well. The *p*-values in (c) and (d) may be anti-conservative (see Materials and Methods, Statistical analyses). The numbers in (d) somewhat depended on the counter, resulting in response curves that may tend towards the negative even when that is not realistic. 2 – 4 genotypes/provenance/treatment, *n* = 2 – 6 plants/genotype/treatment, 75 – 255 measurements/provenance/treatment. The model in (a) is not restricted and extrapolates values below zero. The region of linear growth modeled in (b) is between the dates 32 – 95 DAP and contains 45 – 153 measurements/provenance/treatment.

**
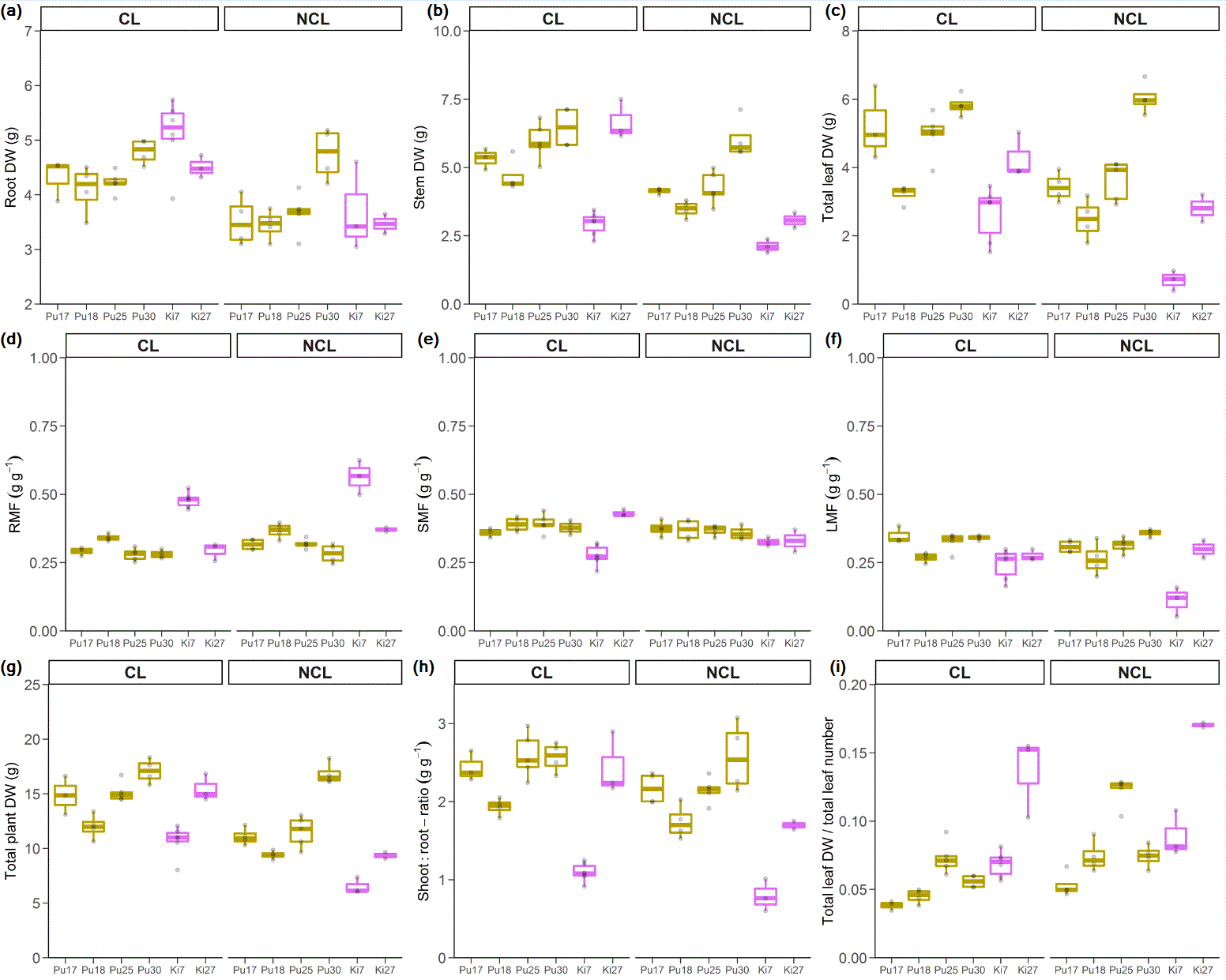
**

**Supplementary Figure S3.** Genotype-level biomass-related data. CL = continuous light, NCL = non-continuous light. Data corresponding to Fig. 4 in text. **(a)** Root dry weight, **(b)** stem dry weight, **(c)** total leaf dry weight, **(d)** root mass fraction, **(e)** shoot mass fraction, **(f)** leaf mass fraction, **(g)** total plant dry weight, **(h)** shoot:root ratio (shoot-part including leaves) and **(i)** total leaf dry weight divided by total leaf number. Boxplots show distribution of raw data (whiskers denote largest/smallest observation less/greater than or equal to upper/lower hinge + 1.5 * IQR). *n* = 2 – 6 plants/genotype/treatment, 5 – 17 measurements/provenance/treatment.

**
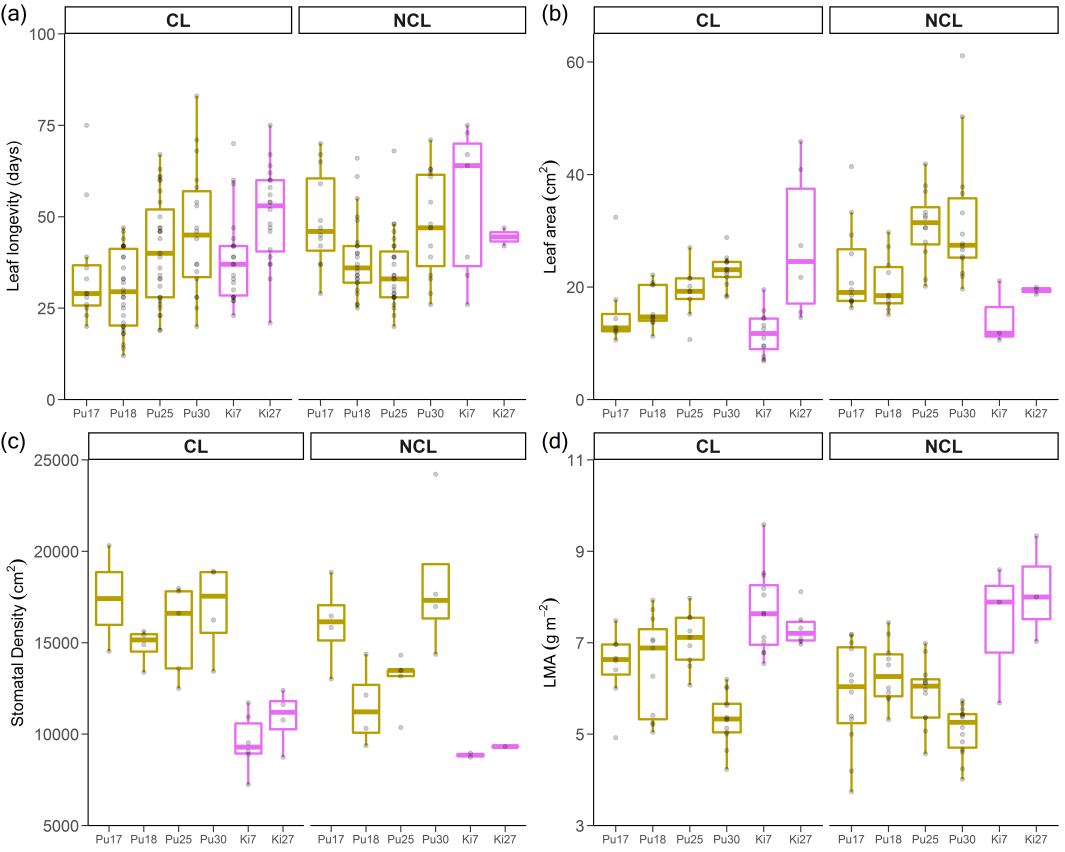
**

**Supplementary Figure S4.** Genotype-level leaf trait data. Data corresponding to Fig. 5 in text. **(a)** Leaf longevity, **(b)** leaf area, **(c)** stomatal density and **(d)** leaf mass per area. Symbols as in Fig. S3. *n* = 1 – 6 plants/genotype/treatment, 3 – 91 measurements/provenance/treatment.


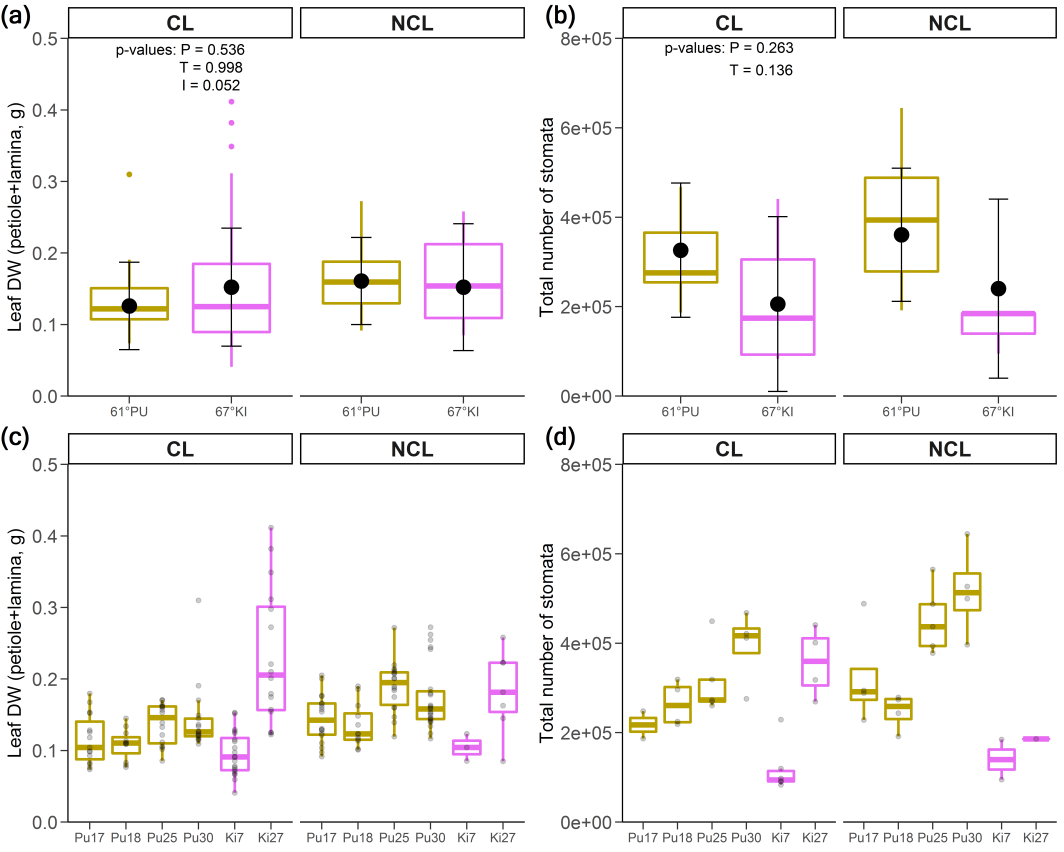


**Supplementary Figure S5. (a)** Leaf dry weight, **(b)** total number of stomata, **(c)** genotype-level data of leaf dry weight and **(d)** genotype-level data of total number of stomata. Black points and error bars are model estimated means and 95% confidence intervals, other symbols as in Figs. S2 and S3. *n* = 1 – 6 plants/genotype/treatment, 3 – 71 measurements/provenance/treatment.

**
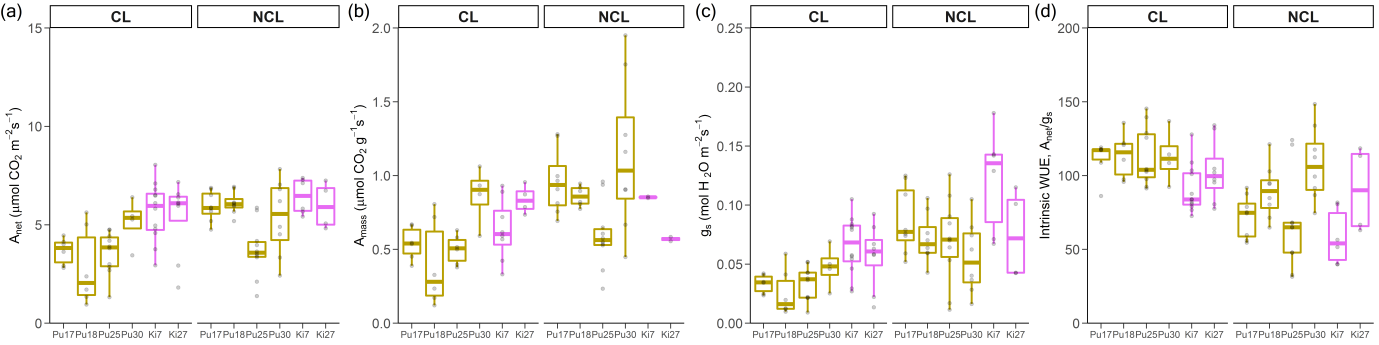
**

**Supplementary Figure S6.** Genotype-level gas exchange data at PPFD = 200 µmol photons m^-2^ s^-1^, data corresponding to Fig. 6 in text. **(a)** Area-based net photosynthesis (A_net_), **(b)** mass-based net photosynthesis (A_mass_), **(c)** stomatal conductance (g_s_) and **(d)** water-use efficiency (WUE). Symbols as in Fig. S3. *n* = 2 – 6 plants/genotype/treatment, 10 – 34 measurements/provenance/treatment. The data is combined from 4 days of measurements, 2 measurements/plant/day (see text for details). The two high values of A_mass_ in 61°PU in the NCL-treatment are not erroneous values – these belong to genotype Pu30 which exhibited both high A_net_ and A_mass_ (but also high spread in A_net_ and A_mass_) and low LMA in NCL.


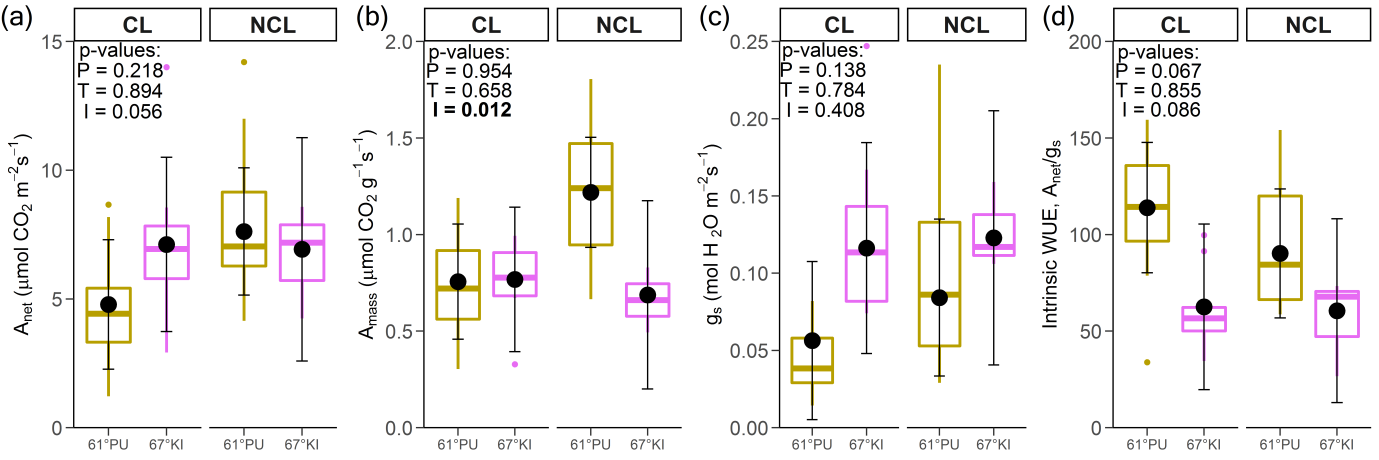


**Supplementary Figure S7.** Gas exchange data at PPFD = 1000 µmol photons m^-2^ s^-1^. **(a)** Area-based net photosynthesis (A_net_), **(b)** mass-based net photosynthesis (A_mass_), **(c)** stomatal conductance (g_s_) and **(d)** water-use efficiency (WUE). Symbols as in Figs. S2, S3 and S5, genotypes not shown for clarity. 2 – 4 genotypes/provenance/treatment, *n* = 1 – 6 plants/genotype/treatment, 3 – 17 measurements/provenance/treatment.

**
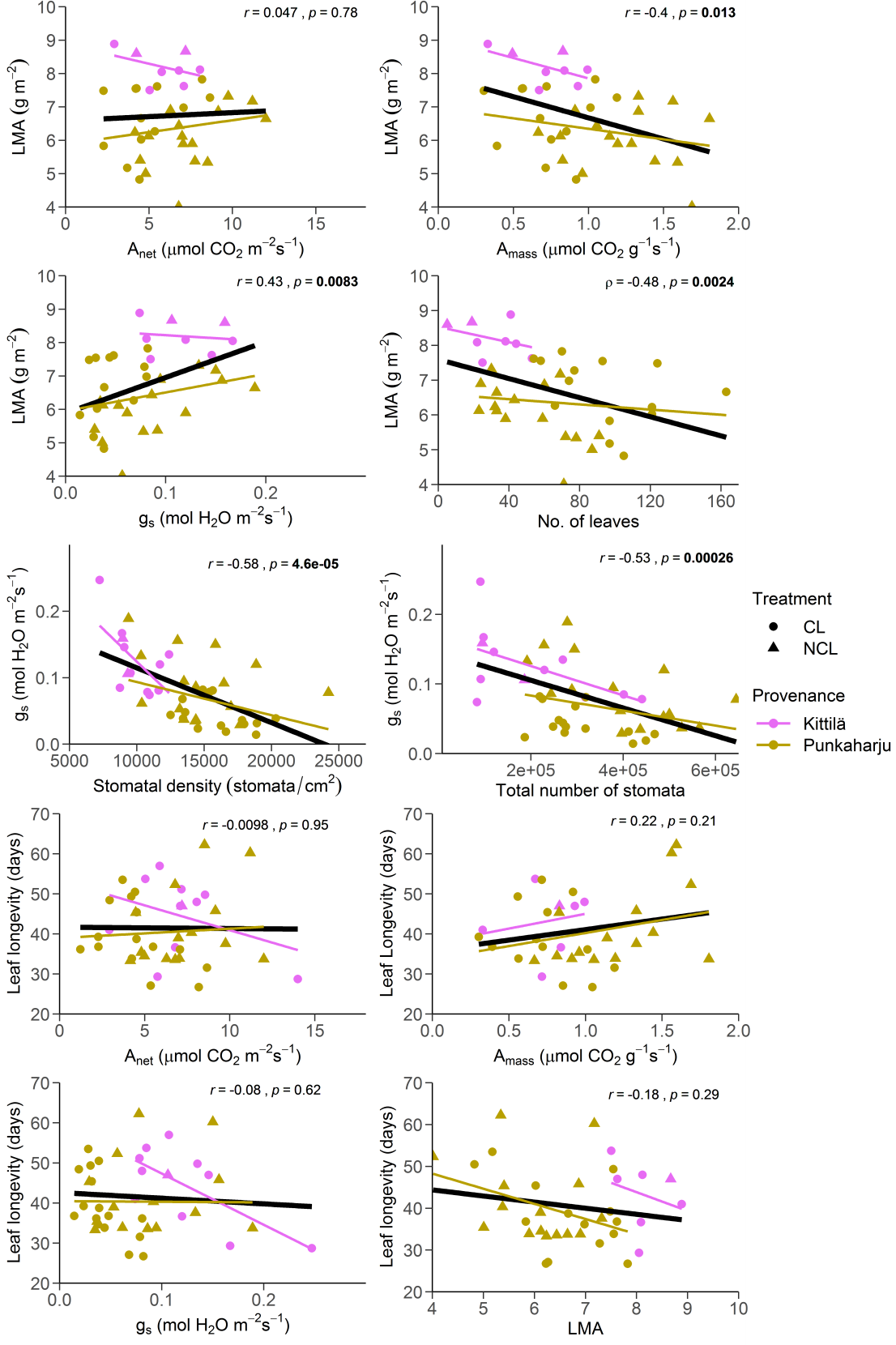
**

**Supplementary Figure S8.** Linear correlations. Dark lines, *r* and *p*-values are the grand correlation across treatments and provenances, colored lines are regression lines based on provenance. Due to small data, we only present the results across treatments, i.e. we are mainly interested in the grand correlation independently of treatment (and provenance). Gas exchange data and stomatal densities are from leaves in group 4 (see Fig. 2 for leaf groups). LMA and leaf longevity are plant averages, LMA is from harvest. The correlation coefficient in LMA vs. total number of leaves is Spearman’s *ρ*, all others are Pearson’s *r*. 1 – 4 genotypes/provenance/treatment, *n* = 1 – 6 plants/genotype/treatment, 1 – 16 measurements/provenance/treatment, depending on each correlation data.
